# *In vivo* evidence for GDP-fucose transport in the absence of transporter SLC35C1 and putative transporter SLC35C2

**DOI:** 10.1016/j.jbc.2023.105406

**Published:** 2023-10-28

**Authors:** Linchao Lu, Shweta Varshney, Youxi Yuan, Hua-Xing Wei, Ankit Tanwar, Subha Sundaram, Mohd Nauman, Robert S. Haltiwanger, Pamela Stanley

**Affiliations:** 1 Department Cell Biology, Albert Einstein College of Medicine, New York, New York, USA; 2 Department of Biochemistry and Molecular Biology, Complex Carbohydrate Research Center, University of Georgia, Athens, Georgia, USA

**Keywords:** GDP-fucose transporter, O-fucose glycans, Notch signaling, skeletal development, gene deletion

## Abstract

*Slc35c1* encodes an antiporter that transports GDP-fucose into the Golgi and returns GMP to the cytoplasm. The closely related gene *Slc35c2* encodes a putative GDP-fucose transporter and promotes Notch fucosylation and Notch signaling in cultured cells. Here, we show that HEK293T cells lacking SLC35C1 transferred reduced amounts of O-fucose to secreted epidermal growth factor-like repeats from NOTCH1 or secreted thrombospondin type I repeats from thrombospondin 1. However, cells lacking SLC35C2 did not exhibit reduced fucosylation of these epidermal growth factor-like repeats or thrombospondin type I repeats. To investigate SLC35C2 functions *in vivo*, WW6 embryonic stem cells were targeted for *Slc35c2*. *Slc35c2*[−/−] mice were viable and fertile and exhibited no evidence of defective Notch signaling during skeletal or T cell development. By contrast, mice with inactivated *Slc35c1* exhibited perinatal lethality and marked skeletal defects in late embryogenesis, typical of defective Notch signaling. Compound *Slc35c1*[−/−]*Slc35c2*[−/−] mutants were indistinguishable in skeletal phenotype from *Slc35c1[−/−]* embryos and neonates. Double mutants did not exhibit the exacerbated skeletal defects predicted if SLC35C2 was functionally important for Notch signaling *in vivo*. In addition, NOTCH1 immunoprecipitated from *Slc35c1*[−/−]*Slc35c2*[−/−] neonatal lung carried fucose detected by binding of *Aleuria aurantia* lectin. Given that the absence of both SLC35C1, a known GDP-fucose transporter, and SLC35C2, a putative GDP-fucose transporter, did not lead to afucosylated NOTCH1 nor to the severe Notch signaling defects and embryonic lethality expected if all GDP-fucose transport were abrogated, at least one more mechanism of GDP-fucose transport into the secretory pathway must exist in mammals.

GDP-fucose, the donor substrate for all fucosyltransferases, is synthesized in the cytoplasm through a *de novo* pathway from mannose, or a salvage pathway that uses fucose from degraded glycoproteins or glycolipids, or from exogenous L-fucose (1, 2, 3, 4, 5). In mammalian cells, GDP-fucose is transported into the lumen of the Golgi apparatus by the GDP-fucose transporter SLC35C1 (6, 7, 8), where it is utilized by various fucosyltransferases (FUTs) (Fig. 1). GDP-fucose is also accessed by an unknown mechanism by POFUT1 and POFUT2 fucosyltransferases that reside in the endoplasmic reticulum (ER) (9, 10). Recent studies have provided evidence for different pools of GDP-fucose in the cytoplasm depending on the source of GDP-fucose from GDP-mannose (*de novo* pathway) or L-fucose (salvage pathway) (4, 5). Mutations of the human GDP-fucose transporter gene *SLC35C1* cause the rare disease leukocyte adhesion deficiency type 2 (LAD II), or congenital disorder of glycosylation SLC35C1-CDG, characterized by severe immunodeficiency, psychomotor defects, delayed mental development, and slow growth (11, 12, 13, 14, 15, 16). SLC35C1-CDG mutations can affect SLC35C1 transport activity or SLC35C1 Golgi localization (17).

**Figure 1:**
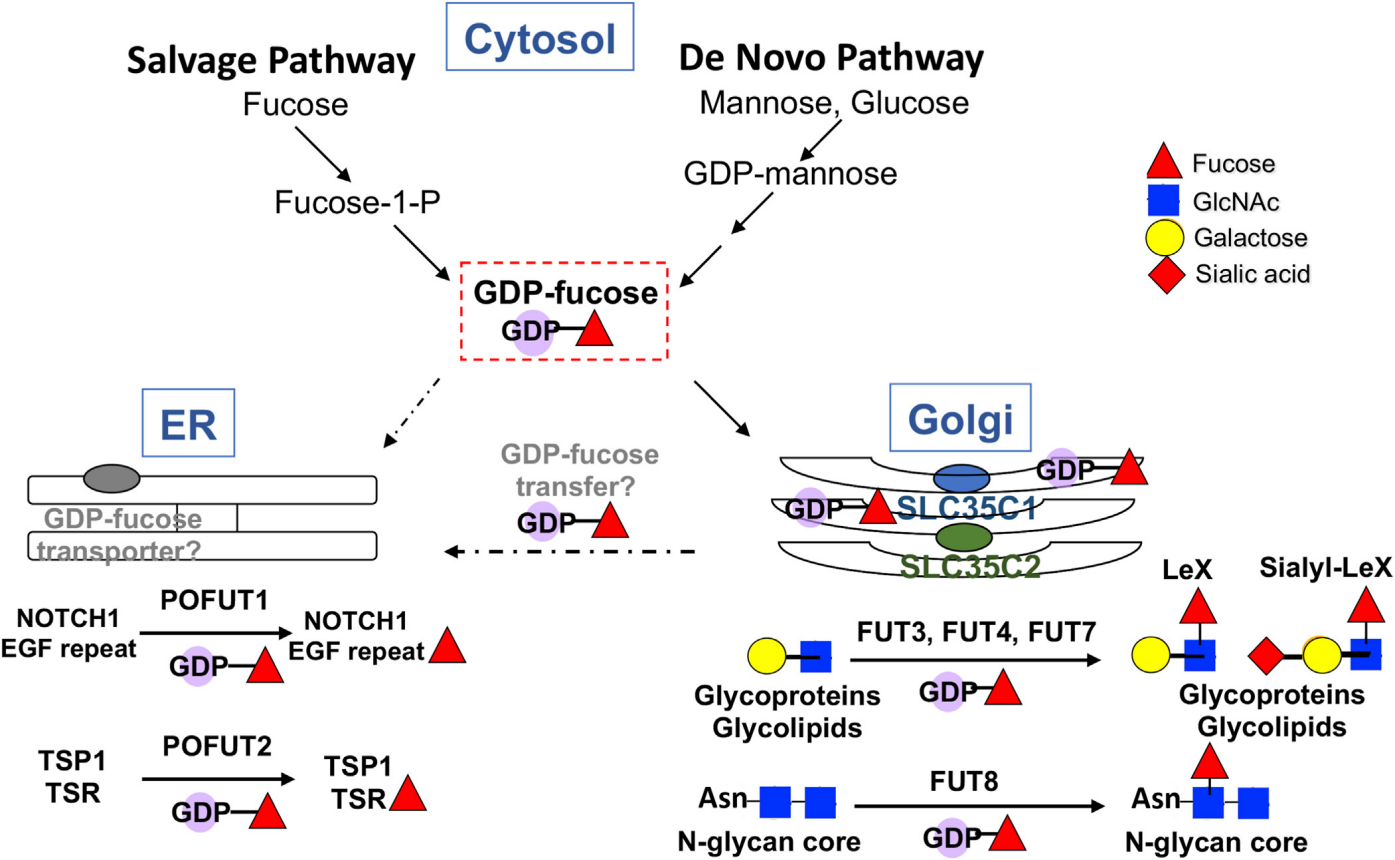
**GDP-fucose synthesis, transport, and utilization.** The diagram summarizes known mechanisms of GDP-fucose synthesis in the cytosol from glucose or mannose *via* a *de novo* pathway from GDP-Man or from fucose *via* a salvage pathway (63). From the cytosol, GDP-fucose is transported into the Golgi by SLC35C1 (*blue oval*). The related protein SLC35C2 (*green oval*) also resides in the Golgi (of rat liver at least) but has not been shown directly to transport GDP-fucose. In this work, we show that elimination of both SLC35C1 and SLC35C2 does not prevent the transfer of fucose to NOTCH1 providing strong evidence for the existence of either an ER GDP-fucose transporter (*gray oval*) like *Efr* in *Drosophila* (see text) or some novel means of GDP-fucose transfer (?) to the ER. POFUT2 also resides in the ER and transfers GDP-fucose to thrombospondin type 1 repeats (TSRs). Transfer of fucose to the core of complex or hybrid N-glycans (by FUT8) or the GlcNAc in lactosamine units in glycans of glycoproteins and glycolipids (by different FUTs) generating LeX or sialyl-LeX epitopes occurs in the Golgi. All fucose residues that are terminal (*i.e.* not further substituted) are recognized by the fucose-binding lectin AAL. See text for further details and references. AAL, *Aleuria aurantia* lectin; ER, endoplasmic reticulum; FUT, fucosyltransferase; LeX, Lewis X.

Mice with a targeted mutation of the *Slc35c1* gene were previously described (18). Homozygous mutants have no *Slc35c1* transcripts in tissues tested and exhibit a similar phenotype to LAD II patients, with complete absence of E- and P-selectin binding to granulocytes and growth retardation (18, 19). *Slc35c1* null mice were born at the expected Mendelian ratio, but exhibited an increased postnatal mortality rate, and only 50% were fertile (18). Based on lectin binding studies, fucosylation of N-glycans and O-glycans in tissues and primary cells from these mice was virtually absent (18). Loss of the fucosylated epitope sialyl-Lewis X led to severely reduced leukocyte adhesion and rolling, reduced trafficking of lymphocytes to inflamed peritoneum, and reduced lymphocyte homing to lymph nodes (19). However, the migration of lymphocytes to spleen and T cell–dependent antibody (Ab) responses were not affected in *Slc35c1*[−/−] mice (19), which provides an explanation for the normal Ab responses observed in LAD II patients (20). Similar to LAD II patients (21), the hypo-fucosylation of primary cells isolated from *Slc35c1* null mice was corrected by supplementation with L-fucose in culture media (18). Residual fucosylation was detected on LAD II leukocytes and fibroblasts (22, 23) and [^3^H]-fucose on immunoprecipitated NOTCH1 was comparable in LAD II and control fibroblasts (24). The fact that neither mice nor humans lacking SLC35C1 exhibit the severe Notch signaling defects observed in the absence of POFUT1 which transfers fucose to Notch receptors (25, 26), suggests the existence of another GDP-fucose transport activity.

The gene *Slc35c2* clusters by sequence with *Slc35c1* and is the most likely candidate for an alternative GDP-fucose transporter in mammals (8, 27). *Slc35c2* has ∼22% to 23% sequence identity and ∼37% to 41% similarity to *Slc35c1* in comparisons from *Drosophila* to human and is highly conserved between species. SLC35C2 localizes to Golgi fractions of rat liver (28). Previous data from this laboratory showed that overexpression of *Slc35c2* decreases synthesis of the fucosylated epitopes LeX and sialyl-Lewis X (Fig. 1) in LEC11B Chinese hamster ovary (CHO) cells (29). A possible explanation is that SLC35C2 directs GDP-fucose to an earlier secretory compartment where, for example, Notch receptors are fucosylated by POFUT1 (9). We previously showed that knockdown of *Slc35c2* in CHO cells reduces NOTCH1 fucosylation and Notch signaling (28). Here, we use cell-based assays to show that, in contrast to knockout of *SLC35C1* in HEK293T cells, inactivation of the *SLC35C2* gene did not significantly reduce fucose addition to a secreted epidermal growth factor–like (EGF) repeat fragment of the NOTCH1 extracellular domain (ECD), nor to thrombospondin type 1 repeats (TSRs) from thrombospondin 1 (TSP1). In addition, knockout of *Slc35c2* in the mouse did not give an observable phenotype, nor did deletion of *Slc35c2* exacerbate the newly identified *Slc35c1* skeletal phenotype. Finally, NOTCH1 was fucosylated in compound *Slc35c1:Slc35c2* null neonates, revealing the existence of another mechanism whereby GDP-fucose is provided to POFUT1 for the transfer of fucose to NOTCH1. A summary of what is known about GDP-fucose synthesis, transport and utilization in the Golgi and the ER is given in Figure 1.

## Results

### SLC35C1 but not SLC35C2 contributes to fucosylation of secreted EGF repeats and TSRs

**Figure 2:**
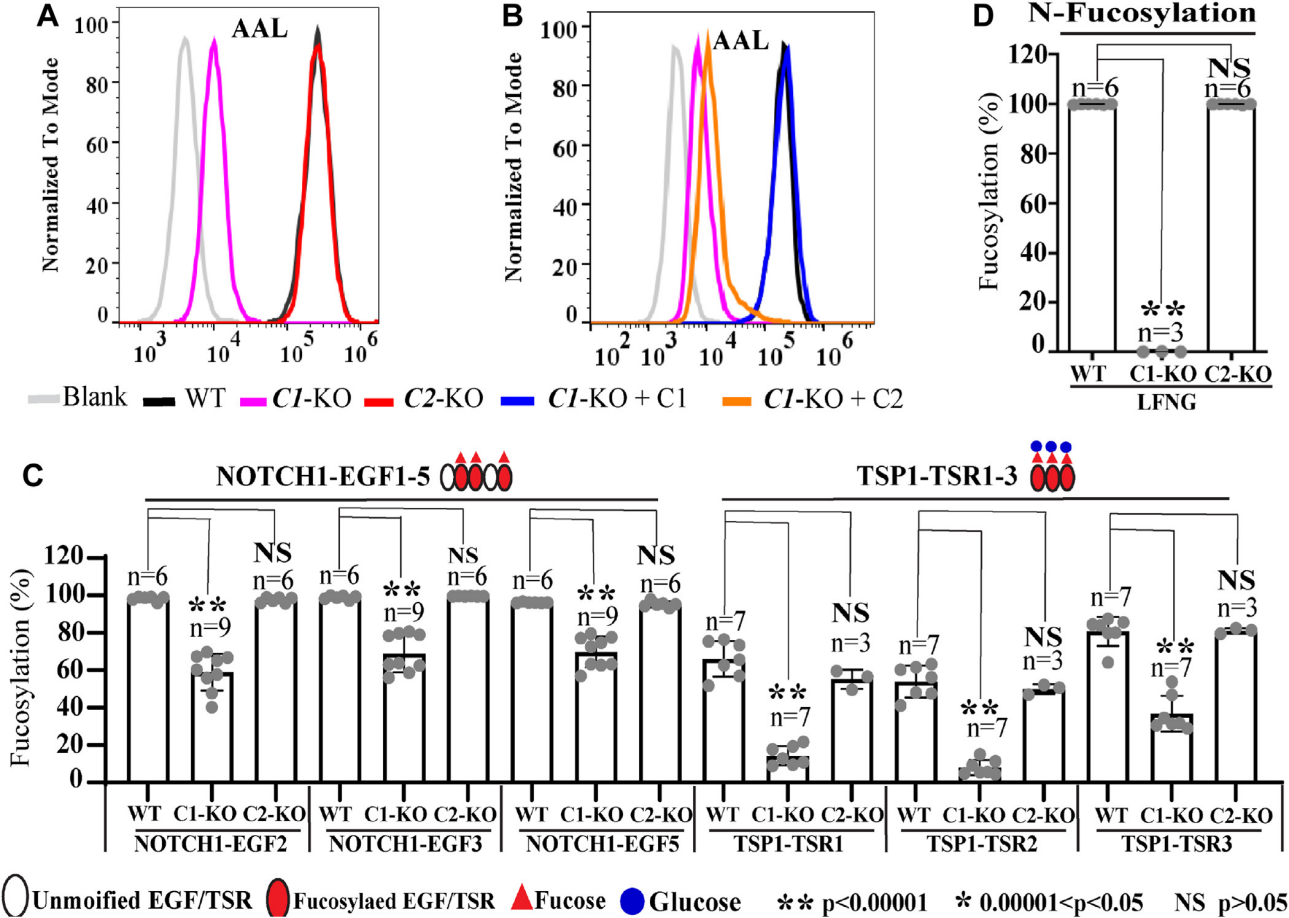
**Deletion of *SLC35C1* in HEK293T cells reduced O-fucosylation of EGF repeats and TSRs but deletion of *SLC35C2* did not.***A*, flow cytometric analysis of fluorescent AAL binding to WT, *SLC35C1*-KO (clone 25.3) and *SLC35C2*-KO (clone 59) HEK293T cells, or WT control (Blank-AAL incubated with 5 mM L-Fucose). The results are representative of three replicates, with mean fluorescence intensity (MFI) normalized to WT (100%). *SLC35C1*-KO was 3.8 ± 0.24% and *SLC35C2*-KO was 94 ± 2.7% of WT. Similar results were obtained with a second clone for each KO (Fig. S1*E*). *B*, AAL binding to *SLC35C1*-KO (clone 25.3) cells expressing a cDNA encoding *SLC35C1* or *SLC35C2*. The results are representative of four replicates, with MFI normalized to WT (100%). *SLC35C1*-KO was 4.8 ± 1.1%, *SLC35C1*-KO+C1cDNA was 90.7 ± 11.6%, and *SLC35C1-KO*+C2cDNA was 6.3 ± 1.4% of WT. Similar results were obtained with a second *SLC35C1-KO* clone (Fig. S1*F*). *C*, NOTCH1 EGF1-5 and TSP1 TSR1-3 were purified from the medium of WT, *SLC35C1*-KO (clone 25.3), or *SLC35C2*-KO (clone 59) cells expressing mouse NOTCH1 EGF1-5-Myc-His_6_, or TSP1 TSR1-3-Myc-His_6_, digested with proteases, and analyzed by nano-LC-MS/MS. The relative abundance of O-fucosylated peptides (percent of total modified and unmodified peptides) from NOTCH1 EGF2, 3, and 5, and TSP1 TSR1, 2, and 3 are plotted. Note that the fucosylated forms of TSRs include both the monosaccharide and disaccharide forms of O-fucose. Relevant MS/MS spectra are in Figs. S2 and S3. *D*, LFNG was purified from the medium of WT, *SLC35C1*-KO (clone 25.3), or *SLC35C2*-KO (clone 59) cells expressing mouse *Lfng*-Myc-His_6_, digested with protease, and analyzed by nano-LC-MS/MS. The relative abundance of N-glycan fucosylated peptide (percent of total modified and unmodified peptides) bearing the only N-glycan on LFNG is plotted. An MS/MS spectrum for this peptide is shown in Fig. S4. A two-tailed assuming unequal variance *t* test was used to determine the statistical significance of fucosylated glycoforms. Error bars are SD. AAL, *Aleuria aurantia* lectin; cDNA, complementary DNA; EGF, epidermal growth factor; LFNG, Lunatic Fringe; MS/MS, tandem mass spectrometry; TSP, thrombospondin; TSR, thrombospondin type 1 repeat.

To determine the ability of SLC35C1 and SLC35C2 to provide GDP-fucose for the fucosylation of secreted EGF repeats 1 to 5 from mouse NOTCH1 or TSRs 1 to 3 from human TSP1, a CRISPR/Cas9 deletion strategy was used (Fig. S1; Table S1). Two independent clones devoid of either SLC35C1 or SLC35C2, respectively, were isolated (Fig. S1). Interestingly, in WT cells, both multitransmembrane proteins ran anomalously at ∼30 kDa, whereas their respective molecular weights are predicted to be ∼40 kDa (Fig. S1, *C* and *D*). Mutant clones were compared with WT HEK293T cells for *Aleuria aurantia* lectin (AAL) binding. AAL is a plant lectin that binds to terminal fucose on the glycans of glycoconjugates (30). Fucose specificity was shown by inhibiting AAL binding to WT HEK293T cells by coincubation in 5 mM L-fucose. Loss of SLC35C1 markedly reduced AAL binding (Figs. 2*A* and S1*E*), and this was corrected by transient transfection of an *SLC35C1* complementary DNA (cDNA) but not by transfection of an *SLC35C2* cDNA (Figs. 2*B* and S1*F*). By contrast, cells lacking SLC35C2 showed no significant reduction in AAL binding (Figs. 2*A* and S1*E*).

A secreted fragment of mouse NOTCH1 ECD including EGF repeats 1 to 5 is an established substrate of POFUT1, and the secreted TSP1 fragment including TSRs 1 to 3 from human TSP1 is an established substrate of POFUT2 (31). To determine if inactivation of *SLC35C1* or *SLC35C2* affected fucosylation of either substrate, reporter cDNAs were transiently expressed in the relevant HEK293T cell lines, tagged protein products were purified by affinity chromatography, and fucosylation was quantitated by mass spectrometry. In cells lacking SLC35C1, a significant reduction in fucosylation was observed at the O-fucose site in each EGF repeat and TSR (Figs. 2*C*; S2 and S3). However, the absence of only SLC35C2 gave no significant reduction in fucosylation compared to WT (Fig. 2*C*). In addition, fucosylation of the complex N-glycan of Lunatic Fringe (LFNG) was >95% lost in cells lacking SLC35C1, but not significantly reduced in cells lacking SLC35C2 (Figs. 2*D* and S4). Thus, SLC35C2 did not contribute to fucosylation of secreted EGF repeat or TSR fragments, nor to fucosylation of the LFNG N-glycan in HEK293T cells. To determine if functions related to fucosylation of full-length NOTCH1 would be affected by SLC35C1 or SLC35C2 *in vivo*, we procured *Slc35c1* mutant mice (18) and deleted the *Slc35c2* gene in the mouse.

### *Slc35c2* gene targeting

**Figure 3:**
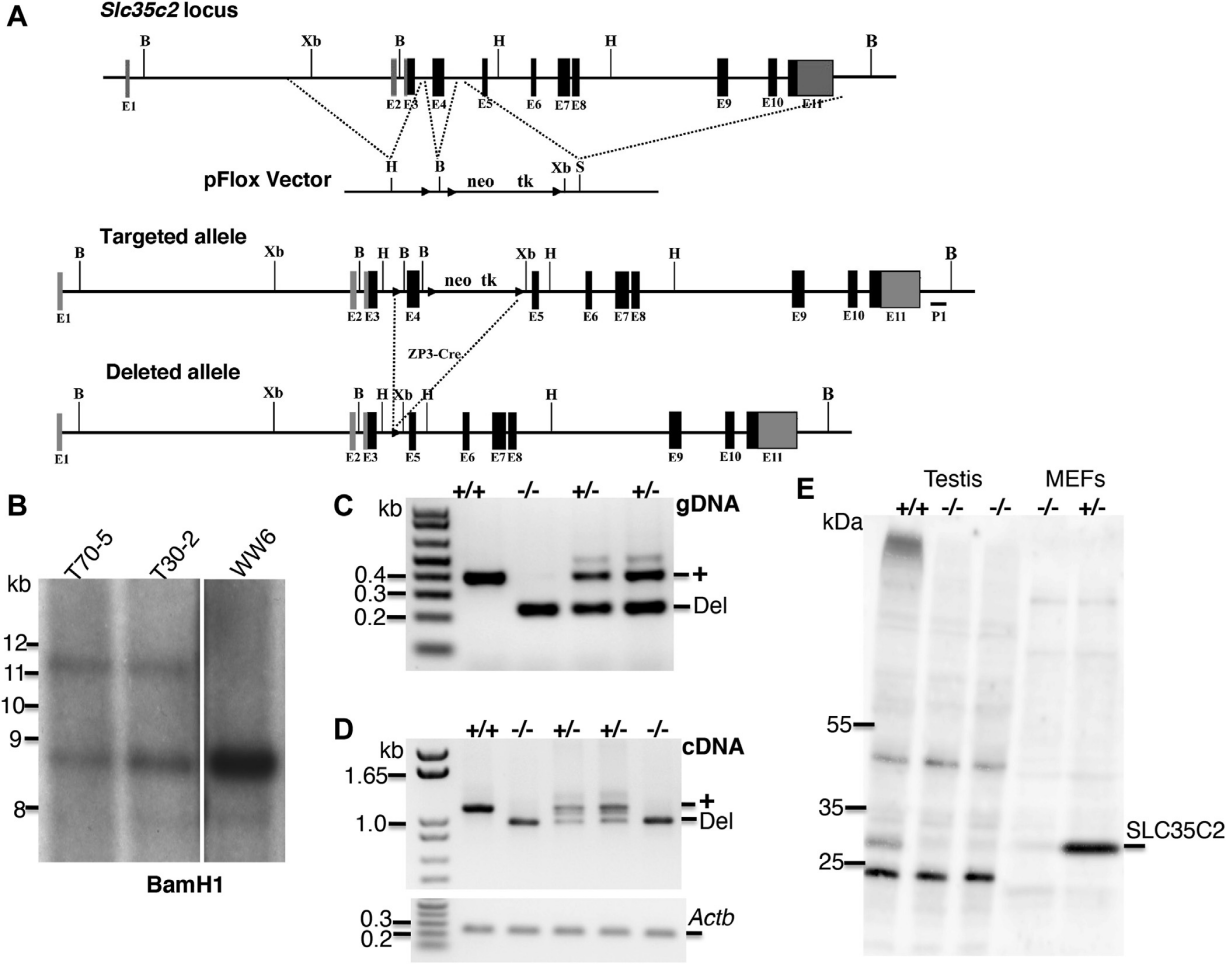
**Targeted deletion of *Slc35c2* in the mouse.***A*, the *Slc35c2* genomic locus of 11 exons was targeted at exon 4 with the pFlox vector that includes a neomycin (neo) and thymidine kinase (tk) cassette and two loxP sites (*black triangles*) that flank ∼800 bp of genomic DNA. The deleted allele was obtained by crossing mice with a targeted *Slc35c2* allele with mice carrying a ZP3-Cre recombinase transgene (64, 65). Thus, exon 4 was removed, leaving one loxP site. *B*, BamHI; H, HindIII; Xb, XbaI; S, SalI. *B*, genomic DNA was extracted from WW6 WT and targeted WW6 ES cells C2PC70-5 and C2P30-2, digested with BamHI, and analyzed by Southern analysis using probe P1 to detect 8.7 kb (WT) and 11.2 kb (Targeted) fragments. *C*, characterization of C2PC70-5 targeted ES cells. PCR genotyping of *Slc35c2* in four pups from a cross between C2PC70-5 heterozygotes. Bands derived from a 390 bp WT allele (+) and a 230 bp deleted allele (Del). *D*, RT-PCR of complementary DNA prepared from mouse liver RNA from 3-week pups using primers within the *Slc35c2* ORF. RT-PCR of actin transcripts served as control. Note the reduced level of truncated mutant transcript (Del) compared with WT transcript (+). *E*, Western blot analysis of mouse testis lysate from *Slc35c2*[+/+] and *Slc35c2*[−/−] males (*left*) and mouse embryo fibroblasts (MEF) derived from *Slc35c2*[+/+] and *Slc35c2*[−/−] embryos. The anti-SLC35C2 antibody was previously described (28). ES, embryonic stem.

The *Slc35c2* gene was targeted in WW6 embryonic stem (ES) cells as described in Experimental procedures (Fig. 3*A*). Southern analysis of genomic DNA (gDNA) revealed that targeted clone T70-5 had a disrupted allele and a WT allele of similar intensity, whereas clone T70-2 did not, indicating partial deletion (Fig. 3*B*). Thus, mice derived from WW6 T70-5 were mated with C57BL/6J to obtain *Slc35c2*[+/−] mice, which were mated to obtain *Slc35c2*[−/−] homozygous mutant mice. Genotyping was performed by PCR of gDNA to detect WT and deleted *Slc35c2* alleles (Fig. 3*C*). Transcripts of WT and mutant *Slc35c2* alleles were characterized by RT-PCR from liver cDNA using primers within the *Slc35c2* ORF (Fig. 3*D*). The truncated *Slc35c2* transcript was readily observed in *Slc35c2*[+/−] and *Slc35c2*[−/−] cDNAs. It was ∼150 bp shorter and of weaker intensity than WT, indicating that the deletion of exon 4 affected the stability of mutant transcripts. The mutant band was purified and the deletion of exon 4 confirmed by sequencing. The truncated mutant transcript is predicted to have a premature stop codon in exon 6 giving rise to a 64-aa peptide, including the first transmembrane domain of SLC35C2. However, Western blot analysis showed that the *Slc35c2* targeted allele and transcripts produced no detectable SLC35C2 protein (Fig. 3*E*).

### Phenotypic changes in *Slc35c2* and *Slc35c1* null mice

**Figure 4:**
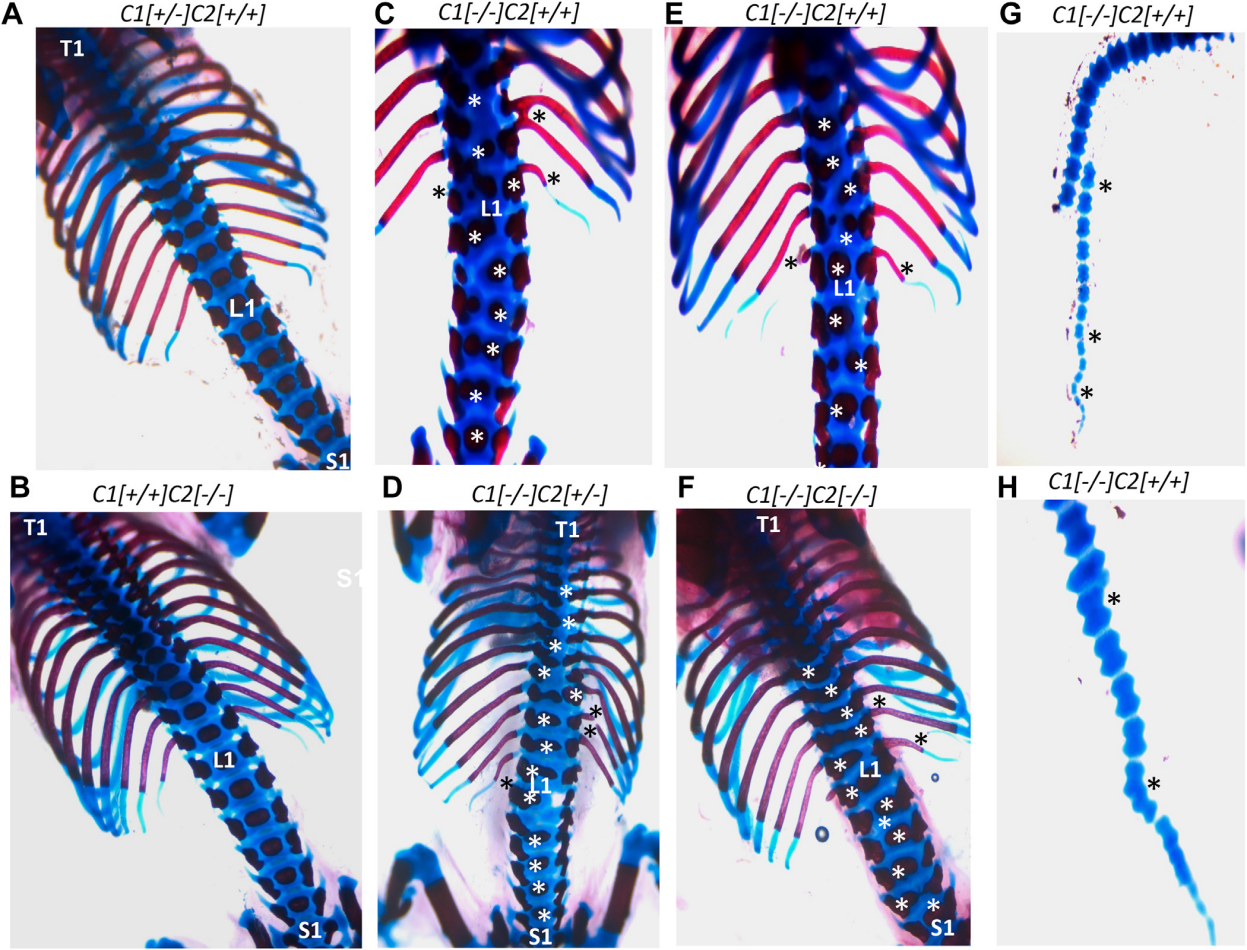
**Skeletal development in *Slc35c2*, *Slc35c1*, and *Slc35c1:Slc35c2* null embryos.***A*–*H,* skeletal preparations from ∼E18.5 embryos of various *Slc35c1* and *Slc35c2* compound genotypes in the C57BL/6J background are shown. Genotypes are indicated by C1 for *Slc35c1* and C2 for *Slc35c2*. Skeletons were stained with *Alcian blue* (cartilage) and *Alizarin red* (bone). T1 is first thoracic vertebra, L1 is first lumbar vertebra, S1 is first vertebra in the sternum. Skeletal abnormalities were similar in mice lacking SLC35C1 and mice null for both SLC35C1 and SLC35C2. *White asterisks* placed near disorganized cartilage reflect bone defects in thoracic and lumbar regions. *Black asterisks* are placed near rib and tail defects giving examples of bifurcated, broken, bent, or missing ribs or tail. Detailed skeletal abnormalities are described for individual mice in Tables S2–S4.

*Slc35c2* homozygous null mice were viable, fertile, and had no obvious developmental defects. Four mating pairs of *Slc35c2*[+/−] X *Slc35c2*[−/−] mice produced 47 pups from 6 litters that reflected the expected Mendelian ratio. *Slc35c2*^*+/−*^ mice were backcrossed to C57BL/6J for >10 generations. To investigate potential changes in skeletal development due to reduced fucosylation of NOTCH1, skeletons were prepared from control and *Slc35c2* null embryos at E18.5 or P0 and microscopically examined. No skeletal abnormalities were detected in homozygous *Slc35c2* null embryos (Fig. 4, *A* and *B*; Table 1).

**Table 1:** Skeletal abnormalities in C57BL/6J *Slc35c1*:*Slc35c2* mutant E18.5 embryos

Mouse genotype	Skeletal Region							
	Thoracic^a^		Ribs		Lumbar		Caudal	
	<4	≥4	Fused	Bent	Split	Misshaped	Fused	Broken
C1[+/+]C2[−/−] (n = 8)	0	0	0	0	0	0	0	0
C1[+/+]C2[+/−] (n = 7)	0	0	0	0	0	0	0	0
C1[+/−]C2[+/−] (n = 7)	0	0	0	0	0	0	0	0
C1[+/−]C2[−/−] (n = 10)	0	0	0	0	0	1	0	0
C1[−/−]C2[+/+]^b^ (n = 9)	4	5	2	9	8	9	2	1
C1[−/−]C2[+/−]^c^ (n = 3)	1	2	1	3	3	3	0	0
C1[−/−]C2[−/−]^d^ (n = 5)	2	3	3	5	5	5	5	0

Details of vertebral abnormalities in each mouse are given in Tables S2–S4.

a Thoracic abnormalities were observed only in the last five vertebrae (T9–T13). Embryos were divided according to ≥4 or <4 vertebral defects). Number of affected mice in each category is shown.

b Among C1[−/−]C2[+/+] embryos, two had both fused and bent ribs; eight had both split and misshaped lumbar vertebrae; One embryo had both fused caudal vertebrae and a broken tail.

c Among C1[−/−]C2[+/−] embryos, one had both fused and bent ribs; all embryos had split and misshaped lumbar vertebrae.

d Two embryos had both fused and bent ribs; three embryos had both split and misshaped lumbar vertebrae.

T and B cell development are highly sensitive to changes in NOTCH1 signaling (32, 33), and the O-fucose glycans of Notch receptors play important roles in T cell development in thymus (34, 35, 36) and B cell development in spleen (37, 38). Recently, SLC35C2 has been identified as a B cell–specific transporter (39). Nevertheless, mice with inactivated *Slc35c2* were not significantly affected in T cell development in thymus or B cell development in spleen (Fig. S5).

Although *O*-fucosylation of Notch is not apparently reduced in LAD II fibroblasts with a T308R mutation in *SLC35C1* (24), and ligand-induced Notch signaling is fully functional in *Slc35c1*[−/−] mouse embryo fibroblasts (MEFs) and LAD II fibroblasts (28), deletion of the *Slc35c1* homolog in *Drosophila* causes mild Notch pathway defects, which are aggravated by low temperature (40), indicating that *Slc35c1* might play a role in Notch signaling in mammals. We therefore examined *Slc35c1* mutant skeletons.

### Skeletal development in *Slc35c1*[−/−] *versus Slc35c1*[−/−]*Slc35c2*[−/−] neonates

Skeletal development is very sensitive to changes in Notch signaling and extension of fucose attached to NOTCH1 EGF repeats by LFNG is critical (41, 42, 43, 44). *Lfng*[−/−] embryos and pups have a severely deformed axial skeleton and essentially no tail. A hypomorphic *Pofut1* mutation reducing *Pofut1* transcripts by ∼75% (45) causes a range of skeletal defects. *Slc35c1*[−/−] mice were previously shown to have poor fertility (18), so *Slc35c1*[+/−]*Slc35c2*[+/−] mice were mated to produce *Slc35c1*[−/−]*Slc35c2*[−/−] mutants. No *Slc35c1*[−/−]*Slc35c2*[−/−] pups were born in six mating pairs that gave 17 litters of 124 pups, although seven *Slc35c1*[−/−] pups in combination with either *Slc35c2*[+/+] or *Slc35c2*[+/−] were born. To increase the numbers of double mutant mice, intercrosses between *Slc35c1*[+/−]*Slc35c2*[−/−] mice were performed. Four *Slc35c1*[−/−]*Slc35c2*[−/−] pups were born from six mating pairs that gave 76 pups. The double mutant pups exhibited severe growth retardation and early death like *Slc35c1*[−/−] pups described previously (18). Thus, analyses were mostly performed on late-stage embryos or newborn pups. The progeny produced from different crosses of compound mutant mice backcrossed to C57BL/6J or 129X1/SvJ mice are presented in Table S5.

*Slc35c1*[−/−] embryos and pups had easily observable skeletal defects beginning in the ninth thoracic vertebra (Fig. 4, *C*–*E*; Table 1). Details of the abnormalities observed in thoracic and lumbar vertebrae, ribs, sternum, and tail are given in Tables S2–S4. It is apparent that in the C57BL/6J genetic background, *Slc35c1*[−/−] E18.5 embryos had deformed or missing thoracic and lumber vertebrae, sternum, and ribs. Defects in the tail were few. Importantly, there was no enhancement of severity in the skeletons of *Slc35c1*[−/−]*Slc35c2*[−/−] double mutants compared to *Slc35c1*[−/−] progeny (Fig. 4*F*; Table 1; Tables S2–S4). This was also the case in double mutants generated after eight backcrosses to the 129X1/SvJ genetic background (Table S5). Few skeletal defects were observed in posterior lumbar vertebrae or ribs in *Slc35c1*[−/−]*Slc35c2*[−/−] 129X1/SvJ embryos, a phenotype considerably milder than that of double mutants on a C57BL/6J background (Table S6). Therefore, there was no indication from these experiments that removal of *Slc35c2* exacerbated the defective skeletal phenotype of *Slc35c1*[−/−] embryos or pups.

### NOTCH1 carries fucose in *Slc35c1*[−/−]*Slc35c2*[−/−] liver and lung

**Figure 5:**
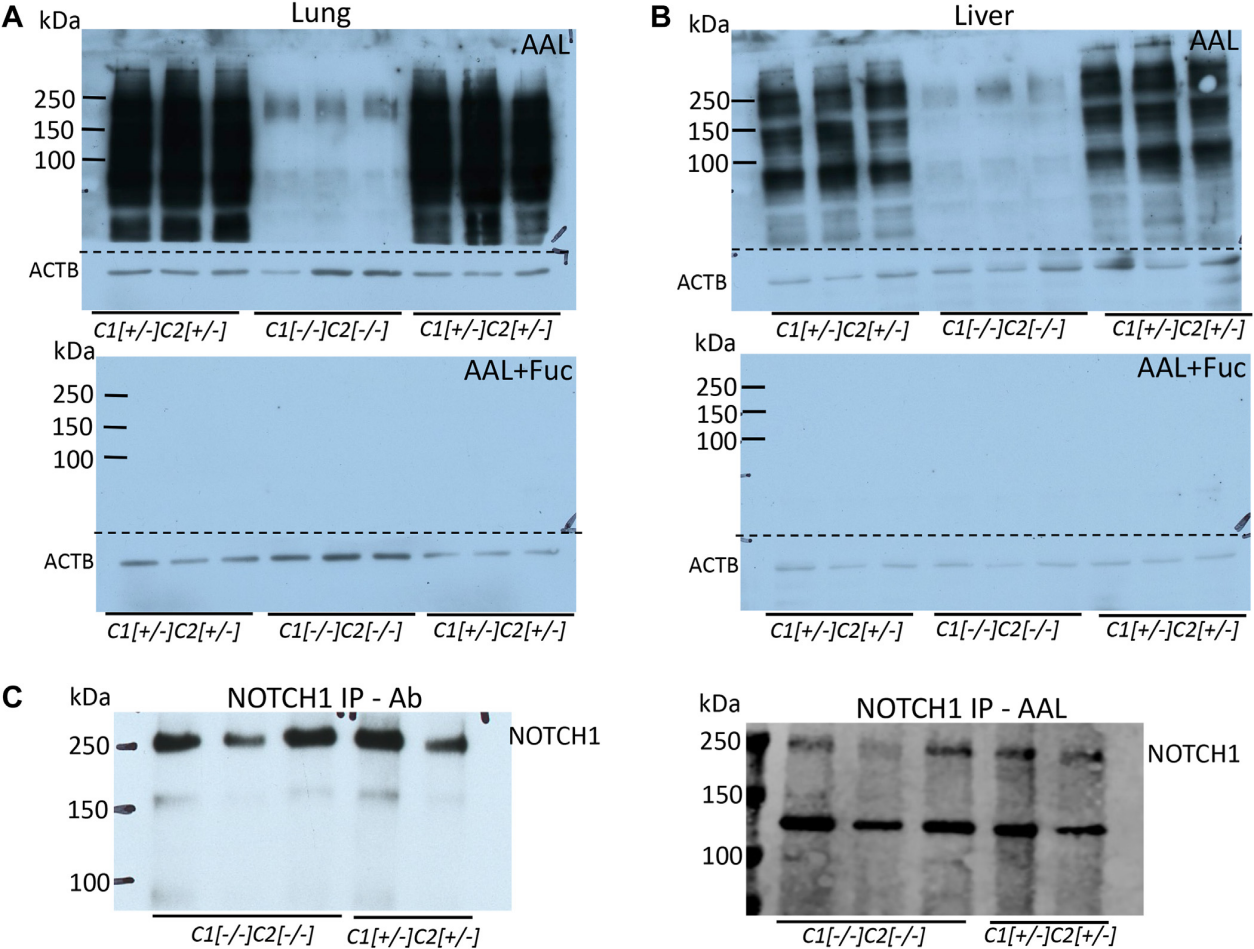
**Fucosylation of NOTCH1 in *Slc35c1:Slc35c2* null lung.***A* and *B*, portions of lung or liver from nine 28 day pups of the indicated genotypes were solubilized as described in Experimental procedures and proteins analyzed by lectin blot using biotinylated AAL alone or biotinylated AAL in the presence of 0.5 M fucose. Antibody to ACTB detected relative loading of each sample. The *dotted line* shows where the blot was cut prior to incubation with respective antibodies. *C*, NOTCH1 was immunoprecipitated from lung lysate of 28 day pups of the indicated genotype as described in Experimental procedures and Fig. S6. Antibody to NOTCH1 ECD detected immunoprecipitated NOTCH1 (NOTCH1 IP+Ab) and NOTCH1+AAL detected immunoprecipitated, fucosylated NOTCH1. AAL, *Aleuria aurantia* lectin; ECD, extracellular domain.

The skeletal changes observed in *Slc35c1*[−/−] and *Slc35c1*[−/−]*Slc35c2*[−/−] mice were consistent with a reduction in Notch signaling during somitogenesis. To determine if NOTCH1 in *Slc35c1*[−/−]*Slc35c2*[−/−] tissues carried O-fucose, cell free extracts of liver and lung from *Slc35c1*[−/−]*Slc35c2*[−/−] and control mice were compared for binding of the fucose-binding lectin AAL. Control mouse liver and lung extracts exhibited AAL binding over a wide range of molecular weights as expected (Fig. 5). AAL binding was essentially prevented by coincubation with 0.5 M L-fucose reflecting fucose specificity. Extracts from *Slc35c1*[−/−]*Slc35c2*[−/−] mice had minimal AAL binding activity (Fig. 5, *A* and *B*), showing that most glycoproteins had no terminal fucose residues. However, the high-molecular weight, AAL-positive bands in double mutant extracts migrated at the position expected for NOTCH1 and NOTCH1 ECD. To determine if O-fucose was present on NOTCH1, NOTCH1 was immunoprecipitated from several independent *Slc35c1*[−/−]*Slc35c2*[−/−] lung extracts and analyzed for AAL binding by lectin blotting. The affinity purification of one preparation is shown in Fig. S6. Immunoprecipitations from independent lysates showed that NOTCH1 from lungs lacking both SLC35C1 and SLC35C2 bound AAL and therefore carried fucose (Fig. 5*C*).

### Expression of related SLC35 transporters in *Slc35c1*[−/−]*Slc35c2*[−/−] mice

**Figure 6:**
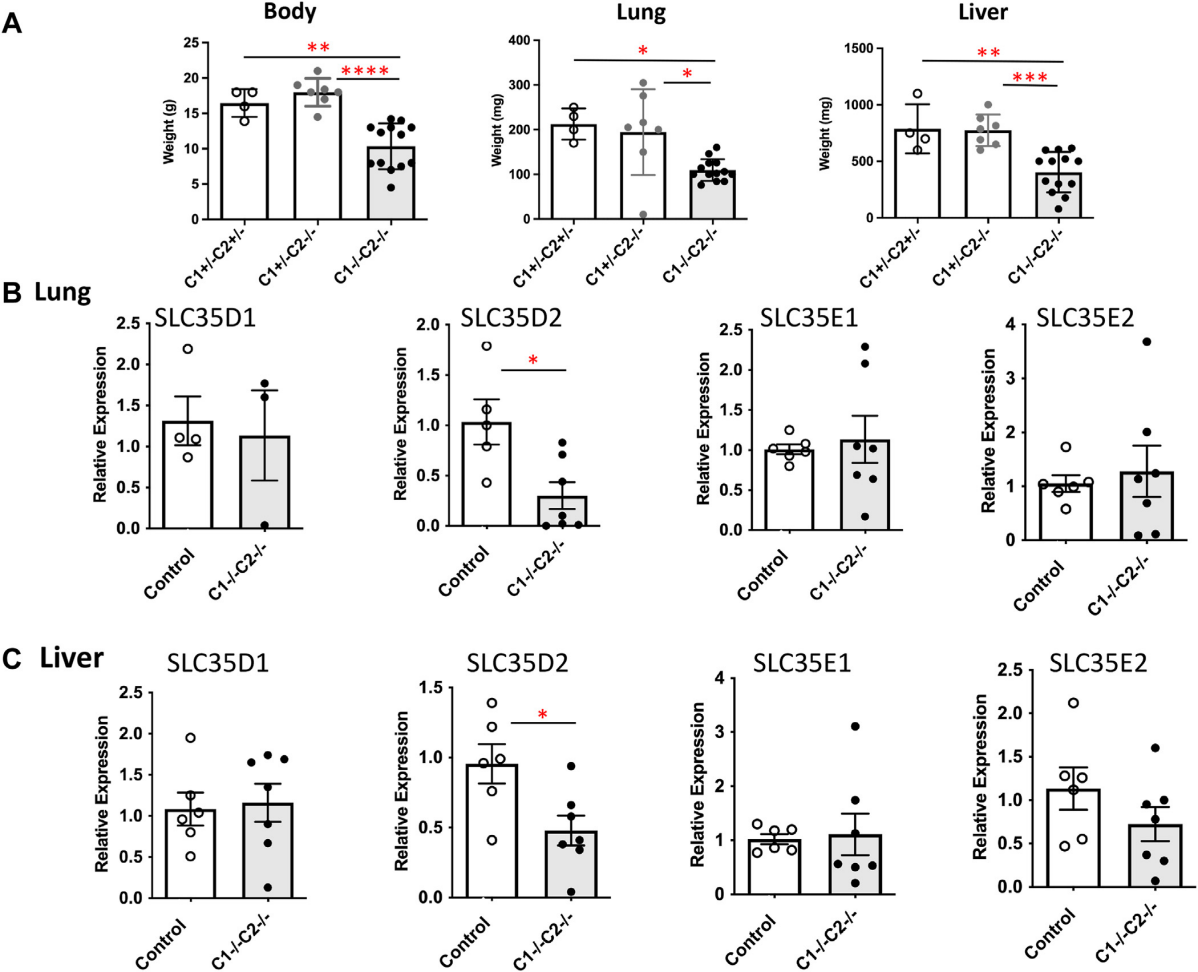
**Expression of related *Slc35* genes in *Slc35c1:Slc35c2* null lung and liver.***A*, body, lung, and liver weights of 28-day pups of the indicated genotype. *B* and *C,* relative expression was determined by comparing *Gapdh* and *Actb* expression with candidate transporter genes by real time quantitative reverse transcription polymerase chain reaction as described in Experimental procedures. Control pups were *Slc35c1*[+/−]*Slc35c2*[+/−] or *Slc35c1*[+/−]*Slc35c2*[−/−] (Control) compared to *Slc35c1*[−/−]*Slc35c2*[−/−] double null pups. Error bars are SEM. *p* < 0.05 (∗), *p* < 0.01 (∗∗), *p* < 0.005 (∗∗∗), and *p* < 0.0001 (∗∗∗∗) determined by two-tailed Student’s *t* test with Welch’s correction.

There are 27 SLC35 genes in mammals only seven of which are proven to transport one or more nucleotide sugars (27). An SLC35 transporter other than SLC35C1 or SLC35C2 may provide GDP-Fuc for POFUT1 in the ER because in double mutant *Slc35c1:Slc35c2* null mice NOTCH1 is fucosylated (Fig. 5). We hypothesized that expression of such an *Slc35* gene might be increased in *Slc35c1:Slc35c2* double null tissues. We used PHI-BLAST to determine genes related to *Slc35c1* and *Slc35c2* and found the most related genes in terms of sequence identity/similarity were *Slc35d1* (23%), *Slc35d2* (26%), *Slc35e1* (27%), and *Slc35e2* (24%), all of which are expressed in developing lung and liver. Top candidates based on DELTA-BLAST were the same four with *Slc35e1* again being most related at 28%. PSI-BLAST gave *Slc35e1* as the only candidate at 27% related. We therefore determined the expression level of these genes in lung and liver from P28 pups. These double mutant pups were smaller than littermates and lungs and livers were similarly reduced in weight (Fig. 6*A*). Quantitative RT-PCR of *Slc35* transcripts for E1, E2, D1, and D2 showed that only *Slc35d2* transcript levels were significantly changed, and they were reduced rather than increased in *Slc35c1:Slc35c2* null mutants compared to controls (Fig. 6*B*).

## Discussion

In this paper, we show that HEK293T cells lacking the GDP-fucose transporter SLC35C1 exhibited significantly reduced O-fucosylation of NOTCH1 EGF repeats 2, 3, and 5 in the context of a secreted fragment of NOTCH1 ECD, although considerable fucosylation remained. We report for the first time that *Slc35c1* null embryos and neonates had skeletal defects consistent with reduced Notch signaling during somitogenesis. The *Slc35c1*[−/−] skeletal phenotype was similar to mice homozygous for a hypomorphic *Pofut1* allele (*Pofut1*^*cax/cax*^) expected to have reduced fucosylation of POFUT1 EGF substrates, including NOTCH1 (45). Less O-fucose on NOTCH1 means fewer sites for the addition of GlcNAc to Fuc-O-EGF by LFNG. Perinatal pups or embryos lacking *Lfng* have very severe skeletal defects in all skeletal regions and essentially no tail (41, 42). Both *Slc35c1* and *Slc35c1:Slc35c2* null pups and *Pofut1*^*cax/cax*^ mice had a tail and appear to have sufficient GlcNAc-Fuc-O-EGF for anterior skeletal development to proceed well. However, they all have defects in the posterior regions of the skeleton. The severity of skeletal defects in these mouse models varies with genetic background (44, 45).

The conclusion that skeletal defects in *Slc35c1* null mice reflect reduced Notch signaling during somitogenesis is supported by experiments in *Drosophila.* The *Drosophila Slc35c1* homolog *Gfr* has 47% identity and 65% similarity to the mouse homolog and is colocalized with Golgi markers in wing disc cells (40). GDP-fucose transport activity of *Drosophila* Gfr was demonstrated using yeast microsomes. A null mutant of *Drosophila Gfr* has mild Notch signaling defects. The mutants exhibit a Notch phenotype in the wing and reduced expression of the Notch target gene *Wg* at 18 °C. Ectopic expression of Fringe rescues the wing phenotype and ectopic *Wg* expression at 18 °C, indicating that Fringe-dependent Notch signaling is deficient in *Gfr* mutants. Moreover, reducing *Slc35c1* transcripts by shRNA in C2C12 cells causes decreased ligand-induced Notch signaling in a coculture assay (40). However, residual Notch signaling and fucosylation in all these experiments indicate the existence of a second GDP-fucose transporter. Such an activity was identified in *Drosophila and* termed *Efr*. This GDP-fucose transporter is localized to the ER and supports Notch signaling (46). Its homolog in mammals is *Slc35b4* which, however, does not transport GDP-Fuc in a yeast microsome assay (47).

By contrast to the reduced O-fucosylation and skeletal defects observed with inactivation of *Slc35c1* in the mouse reported here, removal of *Slc35c2* had no apparent effects on skeletal development in the mouse. Neither did removal of *SLC35C2* from HEK283T cells alter fucosylation of NOTCH1 nor TSP secreted reporter molecules. This was unexpected given that reduced Notch signaling and O-fucosylation were observed following knockdown of *Slc35c2* in CHO cells (28). An explanation for this discrepancy could be that compensatory mechanisms (including a third GDP-fucose transporter activity) that rescue effects of *Slc35c2* deletion on Notch signaling exist in the mouse and HEK293T cells but not in CHO cells. For example, genetic differences between mouse strains convert the hypomorphic *Notch1[12f]* mutation that causes mild Notch signaling defects (34) into an embryonic lethal, Notch signaling-defective phenotype (48).

Here, we show that loss of both *Slc35c1* and *Slc35c2* in the mouse did not exacerbate the defective Notch signaling observed in *Slc35c1* null mice. Moreover, NOTCH1 from mice lacking both the known and putative GDP-fucose transporters SLC35C1 and SLC35C2 was fucosylated. Similarly, deletion of both *SLC35C1* and *SLC35C2* in different cell lines including HEK293T was recently shown to markedly reduce but not prevent fucosylation of N-glycans and O-GalNAc glycans (5). Thus, it is abundantly clear that another mechanism for providing GDP-fucose to POFUT1 exists. To find the missing activity or activities, systematic deletion of each *Slc35* gene in *Slc35c1[−/−]Slc35c2[−/−]* cells might uncover one that is necessary for NOTCH1 O-fucosylation. Alternatively, a CRISPR screen might reveal a gene encoding GDP-fucose transporter activity or a chaperone that allows SLC35C2 to transport GDP-Fuc efficiently. For example, LYSET, a small membrane protein is an essential stabilizer of the phospho-GlcNAc-transferase of lysosomal hydrolases in the Golgi membrane (49, 50). A member of another transporter family, perhaps related to SLC35C1 or SLC35C2 in structure rather than sequence, may be found to transport GDP-fucose. Other possibilities for GDP-fucose transport reside in the major facilitator superfamily (51), a family of secondary active transporters. We hypothesized that one of the four most related *Slc35* transporter genes might be upregulated to compensate for the loss of *Slc35c1* and *Slc35c2* in lung or liver (Fig. 6). However, this did not consistently occur. Unexpectedly, we found that *Slc35d2* transcripts were reduced in *Slc35c1[−/−]Slc35c2[−/−]* lung and liver, presumably by some effect on transcription, potentially related to reduced Notch signaling. SLC35D2 transports UDP-Glc and UDP-GlcNAc but not GDP-Fuc in mammals, although in yeast it transports GDP-Man (52). In fact, the relationship between SLC35 transporters in the Golgi is very complicated due to interactions between apparently unrelated nucleotide sugar transporters in complexes that may also include glycosyltransferases (53, 54, 55). Mice described here that lack both SLC35C1 and SLC35C2 provide an excellent model for establishing the existence of a novel means of GDP-fucose transport.

## Experimental procedures

### Plasmids

The construct expressing EGF repeats 1 to 5 of mouse NOTCH1 ECD (mN1 EGF1-5), TSRs 1 to 3 of human TSP1 TSR1-3, and mouse LFNG in pSecTag (Invitrogen) were described previously (56). To generate the rescue plasmids for *SLC35C1*-KO cells, the pCRISPR-LvSG03 plasmid (57) was digested with restriction endonucleases EcoRI-BamH1 to remove guide RNA expression cassettes and then digested with AgeI-NotI to replace the mCherry coding sequence with a multiple cloning site sequence. Gene-specific primer pairs (Table S1) were used to amplify the coding region of the respective genes from cDNA of HEK293T cells. PCR products were digested with restriction endonucleases EcoRI and EcoRV and ligated into the multiple cloning sites of the rescuing plasmid. The plasmid encoding red florescence protein (RFP), pAd-RFP, was a gift from Tong-Chuan He (Addgene plasmid # 12520; http://n2t.net/addgene:12520; RRID:Addgene 12520).

### Generation of mutant HEK293T cells

*SLC35C1*-KO and *SLC35C2*-KO cells were generated using modified CRISPR/eSpCas9 plasmids (57). Each plasmid encoded the eSpCas9 nuclease, mCherry, a puromycin resistance gene, and a gene-specific guide RNA. HEK293T cells in a 6-well dish were transfected with 3 μg each of two gene-specific plasmids encoding guide RNAs (Table S1) using 10 μl Lipofectamine 2000 transfection reagent (Invitrogen, Cat:11668-019) according to the manufacturer's protocol. After 96 h in culture, single mCherry-positive cells were sorted with a Bio-Rad S3 Sorter by the CTEGD Cytometry Shared Resource Laboratory, UGA, and single cells were added to 96-well plates by limiting dilution. Cells were cultured for 3 weeks in full Dulbecco's modified Eagle's medium (DMEM) with high glucose, 10% bovine calf serum (VWR Cat:10158-358), 1% penicillin and streptomycin (Lonza, Cat:17-602F), and 25% 0.2 μm-filtered 3-day conditioned HEK293T medium. GDNA was isolated from individual clones and WT cells. Sequences flanking the guide RNA were amplified using Hot Start Taq DNA Polymerase (NEB, Cat: M0481L), PCR products were denatured, reannealed with denatured PCR products from WT, and digested with T7 endonuclease I (NEB, Cat:M0302L) according to the manufacturer’s protocol. PCR products from candidate clones were ligated into pGEM-T Easy Vector System (Promega, Cat:A1360) per the manufacturer’s manual, transformed into *Escherichia. coli* (DH5α), and colonies were isolated. Plasmids were isolated from 8 to 10 colonies and the inserts were sequenced. PCR primer pairs and guide RNAs are listed in Table S1.

### HEK293T flow cytometry binding assays

*A. aurantia* lectin conjugated with fluorescein (AAL-FL) was purchased from Vector Laboratories (Cat: F-1391-1). Hanks' Balanced Salt Solution (HBSS) was purchased from Thermo Fisher Scientific (Cat: 14025076). To detect fucose on cell surface glycoproteins, HEK293T cells were grown in DMEM-high glucose medium supplemented with 10% dialyzed fetal bovine serum (FBS; Sigma Cat: F0392-500ML) and 1% penicillin and streptomycin. Cells were pipetted off a Petri dish, pelleted, and washed once with cold DPBS (Dulbecco's PBS). Cells were incubated with AAL-FL (1:1000) in HBSS with 1% bovine serum albumin (BSA) for 1 h on ice. For negative control, cells were premixed with 5 mM L-Fucose to block AAL binding before adding AAL-FL. After washing with HBSS containing 1% BSA twice, cells were resuspended in HBSS with 1% BSA and subjected to flow cytometry using an Accuri C6 Plus flow cytometer (BD Biosciences). Data were analyzed using FlowJo software (BD biosciences; https://www.graphpad.com). For rescue experiments, WT and *SLC35C1*-KO were transfected with RFP plasmid alone, or cotransfected with rescuing plasmid as the experiment indicated. Cells were prepared 72 h later for flow cytometry and gated on RFP-positive cells.

### Protein production

Proteins were transiently expressed in HEK293T cells and purified following the method described previously (58). Briefly, cells were grown in a 10 cm dish with DMEM medium with high glucose, 10% dialyzed FBS, and 1% penicillin and streptomycin. Cells were transfected with 6 μg plasmid (encoding N1 EGF1-5, TSP1 TSR1-3, or LFNG) using 24 μg polyethyleneimine in 6 ml OPTI-minimum essential medium (Invitrogen cat:31985088). Transfection was scaled up when protein was insufficiently produced. Medium was collected 3 days later, and protein was purified using Ni-NTA beads (Qiagen Cat:30230).

### Mass spectral analysis

Protein digestion and analysis by nano-LC-tandem mass spectrometry was performed as described (58). Briefly, purified mouse NOTCH1 EGF1-5-Myc-His_6_, human TSP1 TSR1-3-Myc-His_6_ or mouse LFNG-Myc-His_6_ proteins were reduced, alkylated, and subjected to in-solution digestion with trypsin and chymotrypsin, or trypsin alone. The resulting peptides were analyzed by a Q-Exactive Plus Orbitrap mass spectrometer (Thermo Fisher Scientific) coupled with an Easy nano-LC HPLC system with a C18 EasySpray PepMap RSLC C18 column (50 μm × 15 cm, Thermo Fisher Scientific). Fucosylated peptides were identified using PMI-Byonic (version 2.10.5; Protein Metrics; https://proteinmetrics.com/byonic/) as a node in Proteome Discoverer (v2.1; https://www.thermofisher.com/us/en/home/industrial/mass-spectrometry/liquid-chromatography-mass-spectrometry-lc-ms/lc-ms-software/multi-omics-data-analysis/proteome-discoverer-software.html). Semiquantitative extracted ion chromatogram of selected ions were generated to compare relative amounts of fucosylated and nonfucosylated peptides of each modified peptide (Figs. S2–S4). The mean and SD of the relative abundance of specific fucosylated peptides were compared between WT and KO HEK293T cells. Technical replicates were averaged to obtain a single value of each biological replicate. The mean and SD were calculated from three or more biological replicates. Student’s *t* test with Welch’s correction was performed with data from WT and KO HEK293T cell line using GraphPad Prism (VER 10.1.0; https://www.graphpad.com/updates/prism-1003-release-notes).

### Gene targeting of mouse *Slc35c2*

WW6 ES cells (59) were cultured on a feeder layer of SNL2 fibroblasts in DMEM (Invitrogen) containing 15% ES-qualified FBS (Gemini Bio-Products), and the following ES-qualified ingredients from Chemicon (Millipore-Sigma): 1000 U/ml leukemia inhibitory factor, 1% penicillin and streptomycin, 2 mM L-Glutamine, 1% 2-mercaptoethanol, 1 mM nonessential amino acids, and 25 mM Hepes. GDNA was prepared using DNAzol (Invitrogen) and used as template to generate an ∼800 bp gDNA fragment including exon 4 of the *Slc35c2* gene and two flanking fragments of ∼2.5 kb (left) and ∼5.9 kb (right) by PCR using Elongase (Invitrogen). The targeted vector was constructed by ligating the three fragments into a pFlox vector from Jamey Marth, (University of California at Santa Barbara) containing a neomycin (neo) and thymidine kinase (tk) cassette. Approximately, 20 μg (∼1 μg/μl) of NotI-linearized targeting vector was electroporated into 2 × 10^7^ WW6 ES cells by Gene Pulser II electroporator (Bio-Rad) using the setting of 400 V with a capacitance of 250 μF. ES cells were selected with 250 μg/ml active G418 (Gemini Bio-Products), and gDNA of survivors was screened by PCR using primer SPS-2 (5′-CTCGAGGTCGATCGACGGTATCGAT) in the pFlox vector and primer GS-4 (5′-CCTTAGGAATTTTACATCTGTATAGCCAGT) that is outside of the 5′ flanking fragment. PCR-positive clones were subjected to Southern analysis using a gDNA probe P1 (outside of the 3′ flanking fragment) generated by PCR using primers PS1059 (5′-AAAGGGAATAATGTCTAGCTCAGG) and PS1060 (5′-AACCAGATTTTACTGGGATGTAGC). Two independent ES cell lines were confirmed: C2PC70-5 and C2P30-2. However, the gDNA sequence of C2P30-2 had an ∼1.1 kb deletion including exon 2, 3, and partial exon 4 of *Slc35c2*. Germ-line transmission was obtained from the two ES cell lines. Mice were mated with ZP3Cre transgenic mice (60) to remove exon 4 of *Slc35c2* and the neo-tk cassette. For RT-PCR, total RNA from mouse liver was extracted using TRIZOL (Invitrogen), and cDNA was produced by SuperScript III First-Strand Synthesis System (Invitrogen). *Slc35c2* PCR was performed using primers PS1248 (5′-ACTTAAGCTTATGGGGAGGTGGGCCCTGGA) and PS1294 (5′-ACTTGGTACCTCACTGTTGTCCCTGGGTCA) in the *Slc35c2* ORF. To obtain chimeric mice, ES cells were injected into blastocysts from C57BL/6J mice obtained from Jackson Laboratories. Transfer of the targeted *Slc35c2* allele was determined by PCR genotyping (primers in Table S7), and a mutant strain was developed on a mixed genetic background before backcrossing to C57BL/6J for at least ten generations and subsequently to 129X1/SvJ for eight generations.

### Genotyping

A small tail tip, toe, or portion of decapsulated mouse testis was incubated in 55 μl of proteinase K solution containing 50 mM Tris (pH 8), 100 mM EDTA, 0.5% SDS, and 60 μg proteinase K (Roche, Millipore-Sigma) at 55 °C overnight. Samples were vortexed and centrifuged at 14,000 rpm for 5 min at RT. Supernatant (∼35 μl) was collected and heated at 95 °C for 10 min to inactivate the proteinase K. double distilled water (70 μl) was added and mixed well. Usually, 0.5 to 1 μl was used for genotyping by PCR using various sources of Taq polymerase. For mice generated from ES line C2PC70-5, PCR genotyping was performed using primers PS1115 (5′- GCC TGG TCC TTC TAT ACT ACT GCT TCT CCA TAG -3′) and PS1324 (5′- GGA AGC TCT GTG AAG CCC AAA GAC G -3′) to amplify the WT allele (390 bp) and primers PS1115 and PS1325 (5′- GGA CGG CGA GCT CGA ATT GAT CC -3′) to amplify the deleted allele (230 bp). The genotype of *Slc35c1* WT and mutant MEFs or mice was determined using primers: GFT-F1 (5′-GCG TTG CAA GTT CAG CCG AG-3′), GFT-R2 (5′-CCG TCG ACG GTA TCG ATA AGC-3′), and GFT-R1 (5′-GTG TGT TGG TCA AGA GTG TAA CCT ATG-3′) as described (18). GFT-F1 and GFT-R1 amplify a 2.3-kb product from the WT *Slc35c1* allele and GFT-F1 and GFT-R2 amplify a 1.8-kb product from the mutant allele.

### Mice

All experiments with mice were approved by the Institutional Animal Care and Use Committee of the Albert Einstein College of Medicine under protocol numbers (20080813, 20110803, 20140803, 20170709, 00001311), and all experiments were performed in accordance with the rules and regulations of the Institutional Animal Care and Use Committee. These experiments are reported in accordance with the ARRIVE guidelines (61). Blastocyst injections and transfer to pseudopregnant females were performed by the Gene Targeting Core of the Albert Einstein Cancer Center. Chimeric mice were mated to C57BL/6J mice (stock 000664) from Jackson Laboratorie, and mice obtained from transmission of a mutant allele were intercrossed. Mice with an inactivating *Slc35c1* mutation (18) were a kind gift of Prof Christian Koerner (deceased). Compound mutant mice were obtained by crossing *Slc35c1*[+/−] mice with *Slc35c2*[+/−] or [−/−] mice. Backcrossing was performed for ten or eight generations by crossing *Slc35c1*[+/−]*Slc35c2*[+/−] mice with C57BL/6J (stock 000664) or 129X1/SvJ (stock 002448) from Jackson Laboratories before intercrossing. Mice were euthanized by asphyxiation in a CO_2_ chamber, followed by cervical dislocation.

### Mouse embryo fibroblasts

*Slc35c2* control and null (MEFs) were obtained by trypsinization of E13.5-E14.5 embryos obtained following timed mating of *Slc35c2* heterozygotes as described (28). Fibroblast outgrowths from trypsinized embryos were cultured in minimum essential medium-alpha (Gibco) containing 10% FBS and 1% penicillin and streptomycin.

### Southern analysis

GDNA (∼10 μg) from WW6 ES cells was extracted by DNeasy Blood & Tissue Kit (Qiagen) according to the manufacturer's instructions and incubated with BamHI at 37 °C overnight. DNA was extracted, electrophoresed on 0.8% agarose gel overnight, transferred to nylon membrane overnight, prehybridized for 1 h in Ultrahyb buffer (Ambion, Thermo Fisher Scientific) at 65 °C, and hybridized for 5 h in Ultrahyb buffer at 65 °C with the d-CTP^32^-labeled P1 probe using Prime-It kit (Stratagene, Millipore-Sigma), After washing under stringent conditions, blots were exposed to films at −80 °C.

### Mouse skeleton analysis

Mouse pups were obtained at birth (P0), or fetuses just prior (∼E18.5), or pups up to 8 days later, sacrificed and prepared for skeletal staining as described previously (62). Briefly, mice were fixed in ethanol after removal of skin, muscle, and internal organs. Cartilage was stained in 150 to 300 μg/ml Alcian Blue (Sigma) dissolved at 1:4 in glacial acetic acid in 95% ethanol for 12 to 48 h. After washing twice in ethanol, soft tissues were dissolved in 2% KOH overnight and bones were stained with 75 μg/ml Alizarin Red S (Sigma) in 1% KOH overnight. Destaining was performed in 1% KOH in 20% glycerol for a week, followed by 20% glycerol in 20% ethanol overnight. Skeletons were stored in 50% glycerol, and 50% ethanol.

### Lung and liver protein extraction

P0 mouse liver and lungs were dissected, weighed, washed in PBS and frozen at −80 °C. Frozen tissue (∼0.2 g) was suspended in 500 uL cold homogenizing buffer (50 mM Tris–HCl pH 7.5, 25 mM KCl, 5 mM MgCl_2_, 1× Protease Inhibitor [Roche 05892791001) and 1× PhosStop [Roche 04906845001] with 1% Triton X-100 (Sigma, Cat.T9284) and 1% IGEPAL CA-630 (Sigma, Cat.13021). Stainless steel beads (1.4 mm, Next Advance) were added and homogenization was performed in a Bullet Blender Storm 24 (Next Advance) at setting eight for 3 min at 4 °C. The homogenate was incubated on ice for 20 min, centrifuged at 5000*g* for 5 min at 4 °C, and the supernatant stored at −80 °C. Protein concentration was determined by the Bradford method (Bio-Rad, Cat. 500-0006).

### Abs and lectins

Polyclonal rabbit Abs to a C-terminal peptide of *Slc35c2* conserved in mouse, rat, and human and affinity purified on an antigen column were described previously (28). Horseradish peroxidase (HRP)-conjugated anti-rabbit IgG was from Jackson ImmunoResearch. Sheep anti-NOTCH1 ECD (R&D Systems, Cat.AF5267) was detected using antigen-purified polyclonal HRP–conjugated donkey anti-sheep IgG (R&D Systems, Cat.HAFO16). Rabbit polyclonal affinity–purified Ab to ACTB C-terminal peptide was from Sigma (Cat.A2066). AAL conjugated to biotin (Vector Labs, Cat.B1395) was detected with Streptavidin IRDye 680 (LiCor, Cat.960-68079) and ACTB was detected with donkey anti-rabbit IRDye 800CW (LiCor, Cat.926-32213).

### T and B cell analyses

To investigate T and B cells development, thymus and spleen from mice of 6 to 8 weeks were collected, weighed, and passed through a 70 μm cell strainer (Falcon). Cell suspensions were incubated with red blood cell lysis buffer (eBiosciences-Thermo Fisher Scientific), washed with fluorescence-activated cell sorting buffer (HBSS) with glucose (Corning, Millipore-Sigma), 1 mM CaCl_2,_ 2% (BSA, fraction V, Sigma), and 0.05% sodium azide, (pH 7.2–7.4), and counted. About 5 × 10^5^ cells were pelleted, incubated with rat-anti-mouse CD16/CD32 Ab (mouse BD Fc block; BD Biosciences) on ice for 15 min, and incubated with FL-labeled mAbs anti-CD4-PE (BD Biosciences), CD8α-FITC (eBiosciences), and B220-APC (BD Biosciences) for 30 min in the dark on ice. After three washes, cells were resuspended in fluorescence-activated cell sorting buffer without BSA and 2 μl of 7-amino-actinomycin D (BD Pharmingen) was added. Cells were subjected to flow cytometry using a FACScan (BD Biosciences) instrument. Cells stained by 7-amino-actinomycin D were excluded. Data were analyzed using FlowJo software (Tree Star Inc).

### Western and AAL blot analyses

For the blots in Figure 3, MEFs (28) in a 6-well dish or decapsulated testis were washed with cold PBS pH 7.4 and lysed in 250 μl passive lysis buffer (Promega) containing Complete EDTA-free protease inhibitor (Roche Applied Science). After 10 min incubation on ice, lysates were centrifuged (6000*g*, 5 min, 4 °C), and the protein concentration of the supernatant was determined by Bio-Rad *Dc* protein assay kit (Bio-Rad). Protein (∼60 μg) was separated by SDS-PAGE and transferred to polyvinylidene fluoride membrane blocked in Tris-buffered saline with Tween (25 mm Tris–HCl, 125 mm NaCl, 0.1% Tween 20, pH 7.4) containing 5% nonfat dry milk (NFDM). Abs were diluted in Tris-buffered saline with Tween/NFDM and incubated with the blot for 16 h at 4 °C (primary Ab) or 2 h at room temperature (RT; secondary Ab). Proteins were visualized using enhanced chemiluminescence (SuperSignal kit; Pierce).

For the blots in Figure 5, ∼20 μg liver or lung protein extract was separated by SDS-PAGE on 10% gels. The gels were transferred to polyvinylidene fluoride membranes (Thermo Fisher Scientific 88518) with wet transfer in 5% methanol in transfer buffer (25 mM Trizma base and 192 mM glycine). The blots were cut horizontally at ∼50 Kda. The top portion was blocked in either TBS/Tween 20 or TBS/Tween20 with 0.5 M Fucose (Sigma) at RT for 1 h before incubation in 1:10,000 dilution of HRP-AAL (15-AAL15-HRP, Genprice Inc) in TBS/Tween 20 with or without 0.5 M fucose for 1 h at RT. They were washed four times with TBS Tween 20 for 5 min and TBS twice for 5 min and exposed to X-ray film. The bottom portion of the membrane was blocked with 5% NFDM in TBS with 0.05% Tween 20 and 0.1% Thimerasol at RT for 2 h before incubation in anti-ACTB Ab (Sigma A2066, 1:2000) at 4 °C for 18 h. After four washes of 5 min with TBS/Tween 20, blots were incubated in a 1:10,000 dilution of HRP-conjugated goat anti-rabbit Ab (Sigma) in 5% NFDM in TBS/Thimerasol for 1 h at RT. After four washes of 5 min with TBS/Tween 20 and two with TBS for 5 min each, blots were exposed to X-ray film.

### Immunoprecipitation of mouse NOTCH1

Mouse lung extract (∼500 μg) was precleared with 100 μl washed Streptavidin-Agarose (Invitrogen S951) in 100 μl TBS on a rotator at 4 °C for 1 h. This was further precleared by adding 10 μl protein G+ agarose (Pierce 22851) at 4 °C for 1 h. Anti-NOTCH1-ECD Ab (2 μg) was added to precleared extracts, incubated overnight at 4 °C on a rotator before 10 μl protein G+ agarose was added for 1 h. The samples were centrifuged at 600*g* for 5 min at 4 °C and the supernatant saved (immunoprecipitate supernatant). The protein G+ agarose was washed three times with 100 μl PBS with 0.1% Tween 20. The agarose was incubated with Laemmli gel loading buffer and heated at 80 °C for 20 min. After centrifugation, the immunoprecipitate was divided between two 7.5% SDS-PAGE gels. After electrophoresis, the gels were transferred to nitrocellulose membranes in 5% methanol as above. The blot to be incubated in anti-NOTCH1 Ab was blocked in 5% NFDM in TBS with 0.05% Tween 20 and 0.1% Thimerasol at RT for 2 h. The blot to be incubated with AAL-biotin was incubated with 5% BSA/TBS Tween 20 at RT for 1 h. Membranes were probed with 0.4 μg/ml anti-NOTCH1 ECD in 3% cold fish gelatin in TBS Tween 20 or 1 μg/ml biotinylated AAL in 5% BSA/TBS Tween 20 and anti-ACTB Ab at 4 °C for 18 h. The blots were washed four times for 5 min with TBS/Tween 20. NOTCH1 Ab was incubated with a 1:5000 dilution HRP-donkey anti-sheep Ab in TBS NFDM, and the blots probed with AAL and anti-ACTB was incubated in a 1:25,000 dilution of streptavidin IRDye 680 and donkey anti-rabbit IRDye 800CW for 1 h at RT. They were washed four times with TBS/Tween 20 and TBS twice. The NOTCH1 blot was exposed to X-ray film, and the AAL blots were visualized in the LiCor Odyssey system.

### Quantitative RT-PCR

Frozen lung or liver from P28 pups (∼50 mg) was homogenized in 1 ml TRIZOL and incubated for 5 min at RT before 200 μl chloroform was added. Tubes were vortexed for 15 s, incubated at RT for 2 to 3 min, and centrifuged at 12,000*g* for 15 min at 4 °C. The aqueous phase was transferred to a new tube, and 500 μl isopropanol was added. Samples were incubated for 10 min on ice and centrifuged at 12,000*g* for 10 min at 4 °C. The RNA pellet was washed once with 1 ml 70% ethanol, air-dried for 5 to 10 min, and dissolved in 50 μl RNase-free water. Samples were placed in 55 °C to 60 °C degree water bath for 15 to 20 min and RNA concentration was determined by Nanodrop. cDNA was prepared from 250 ng RNA using the ReverTra Ace qPCR RT Master Mix with gDNA Remover (Toyobo Research Reagents) following the manufacturer’s protocol. Quantitative RT-PCR was performed in duplicate and relative expression compared to *Gapdh* and *Actb* was determined. Primer sequences are given in Table S7.

### Statistics

Data are presented as mean ± SEM or ± STDEV as noted. Calculations were performed using the unpaired Student’s *t* test calculated by GraphPad Prism (GraphPad Software). Mendelian inheritance was analyzed by the *Chi*-squared test.

## Data availability

All data are included in the manuscript and Supporting information.

## Supporting information

This article contains supporting information.

## Conflict of interest

The authors declare that they have no conflicts of interest with the contents of this article.
