# Supplementary material for: In vivo evidence for GDP-fucose transport in the absence of transporter SLC35C1 and putative transporter SLC35C2
Source: J Biol Chem. 2023 Oct 28;299(12):105406. doi: 10.1016/j.jbc.2023.105406 (PMC10709068; doi:10.1016/j.jbc.2023.105406)
Supplement: Supporting Figures S1–S6 and Tables S1–S7 [file mmc1.docx]

**Supplemental Information**

*In vivo* evidence for GDP-fucose transport in the absence of transporter SLC35C1 and putative transporter SLC35C2

Linchao Lu^1, 3,^ * Shweta Varshney^1, 4,^ * Youxi Yuan^2^, Hua-Xing Wei^1, 5^, Ankit Tanwar^1^, Subha Sundaram^1^, Mohd Nauman^1^, Robert S. Haltiwanger^2^ and Pamela Stanley^1, 6^

^1^ Dept. Cell Biology, Albert Einstein College of Medicine, 1300 Morris Park Ave., New York, NY, 10461. **^2^** Complex Carbohydrate Research Center, Department of Biochemistry and Molecular Biology, University of Georgia, Athens, Georgia, USA

**Supporting Figures S1 to S6**

**Supporting Tables S1 to S7**


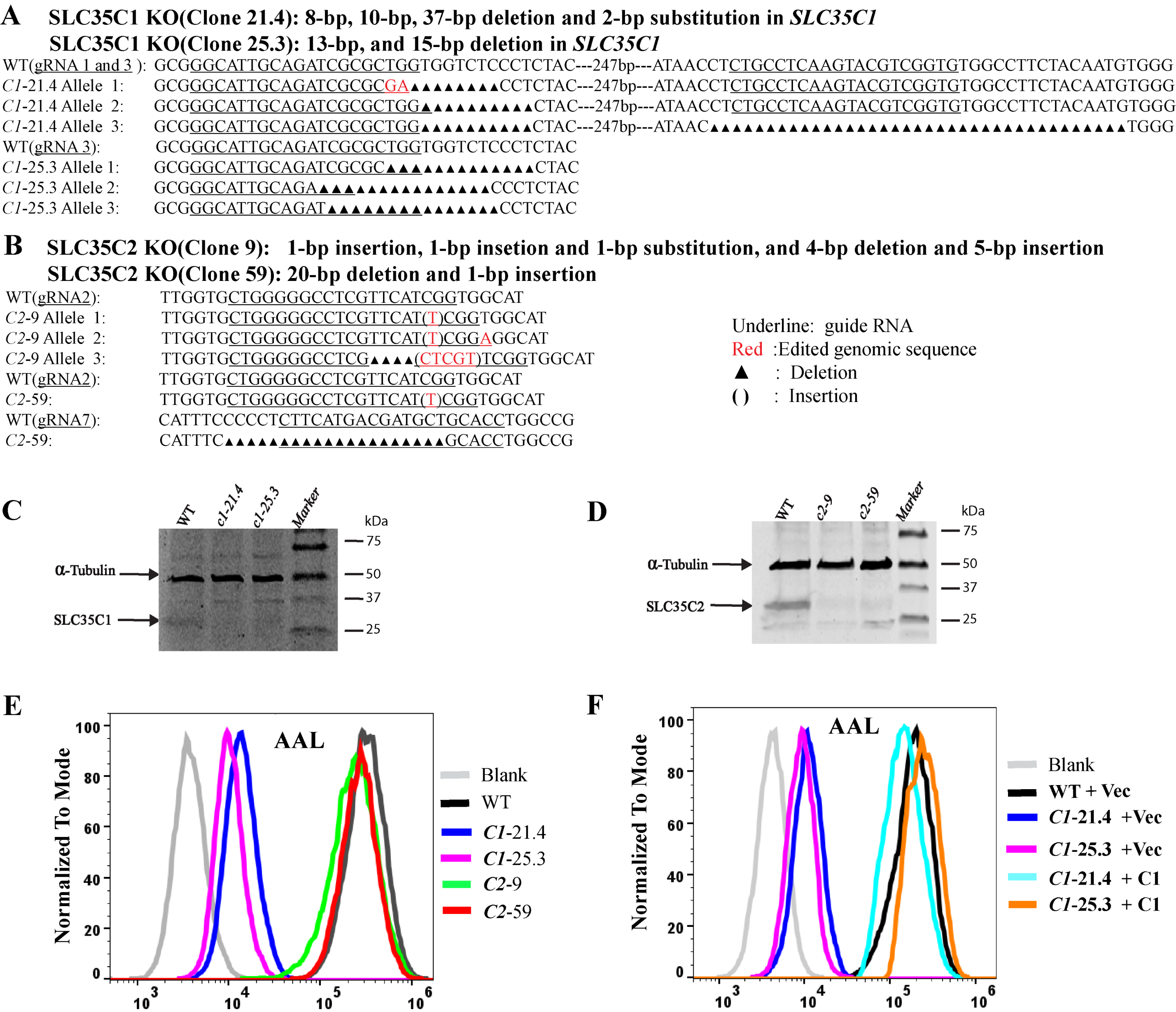


**Figure S1. Generation of *SLC35C1*-KO and *SLC35C2*-KO HEK293T cells using CRISPR-Cas9.** Genomic sequences of *SLC35C1* (**A**) and *SLC35C2* (**B**) amplified from WT and *KO* HEK293T cells. Primers to amplify regions surrounding gRNA sequences (underlined) are in Table S1. No WT sequences were detected in any of the KO cell lines. **A**. Three *SLC35C1 (C1)* alleles were detected in each clone. Sequences from *C1*-KO clone 21.4 revealed frame-shift mutations in all three alleles. Sequences from *C1*-KO clone 25.3 revealed a frame-shift in one allele and different in-frame 15 bp deletions in the other two alleles. Five amino acids were deleted from the first transmembrane sequence which may prevent insertion into the membrane. Western blot analysis (panel C), loss of AAL binding and rescue by co-transfection with WT *SLC35C1*, (panel F) demonstrated loss of SLC35C1 protein and function, respectively. **B.** Genomic sequences of *SLC35C2 (C2)* amplified from WT and *SLC35C2-KO* cells. Sequences from *C2*-KO clone 9 revealed a 1 bp frame-shift mutation in one allele, a 1 bp deletion and 1 bp insertion in another allele, and a 4 bp deletion and 5 bp insertion in the third allele. Sequences from clone 59 revealed frame-shift mutations in one allele of two guide RNA loci. Since no WT sequences were detected, there is likely a large deletion for the other alleles. Western analysis (panel D) demonstrated loss of SLC35C2 protein. **C.** Western blot of total protein lysate from WT and *SLC35C1*-KO clones separated on a 4-20% gradient SDS-PAGE. Protein was transferred onto nitrocellulose membrane and blotted with anti-SLC35C1 antibody (ThermoFisher PA5-64146) and anti-α-tubulin antibody (Sigma-Aldrich, T9026) overnight in the cold room. Membrane was washed three times with TBST and incubated with IRDye 680-conjugated goat anti mouse IgG antibody (LI-COR 1:10,000) and IRDye 800-conjugated goat anti-Rabbit IgG antibody (LI-COR, 1:10,000) diluted in TBST at room temperature for 1 h. After the membrane was washed six times with TBST, the bands were visualized in gray scale using an Odyssey System (LI-COR). **D.** Western blot of total protein lysate from WT and *SLC35C2*-KO clones separated on a 4-20% gradient SDS-PAGE. The method described above was used with anti-SLC35C2 antibody (ThermoFisher A304-624A) and anti-α-tubulin (Sigma-Aldrich, T9026). **E***.* Flow cytometric analysis of fluorescent AAL binding to WT, *SLC35C1*-KO and *SLC35C2*-KO HEK293T cells, or WT control (AAL with additional 5 mM L-Fucose) as described in Experimental Procedures. **F.** AAL binding to *SLC35C1*-KO clones transfected with a plasmid encoding *SLC35C1* (C1) or transfected with empty vector (Vec). This figure is related to

Figure 2.


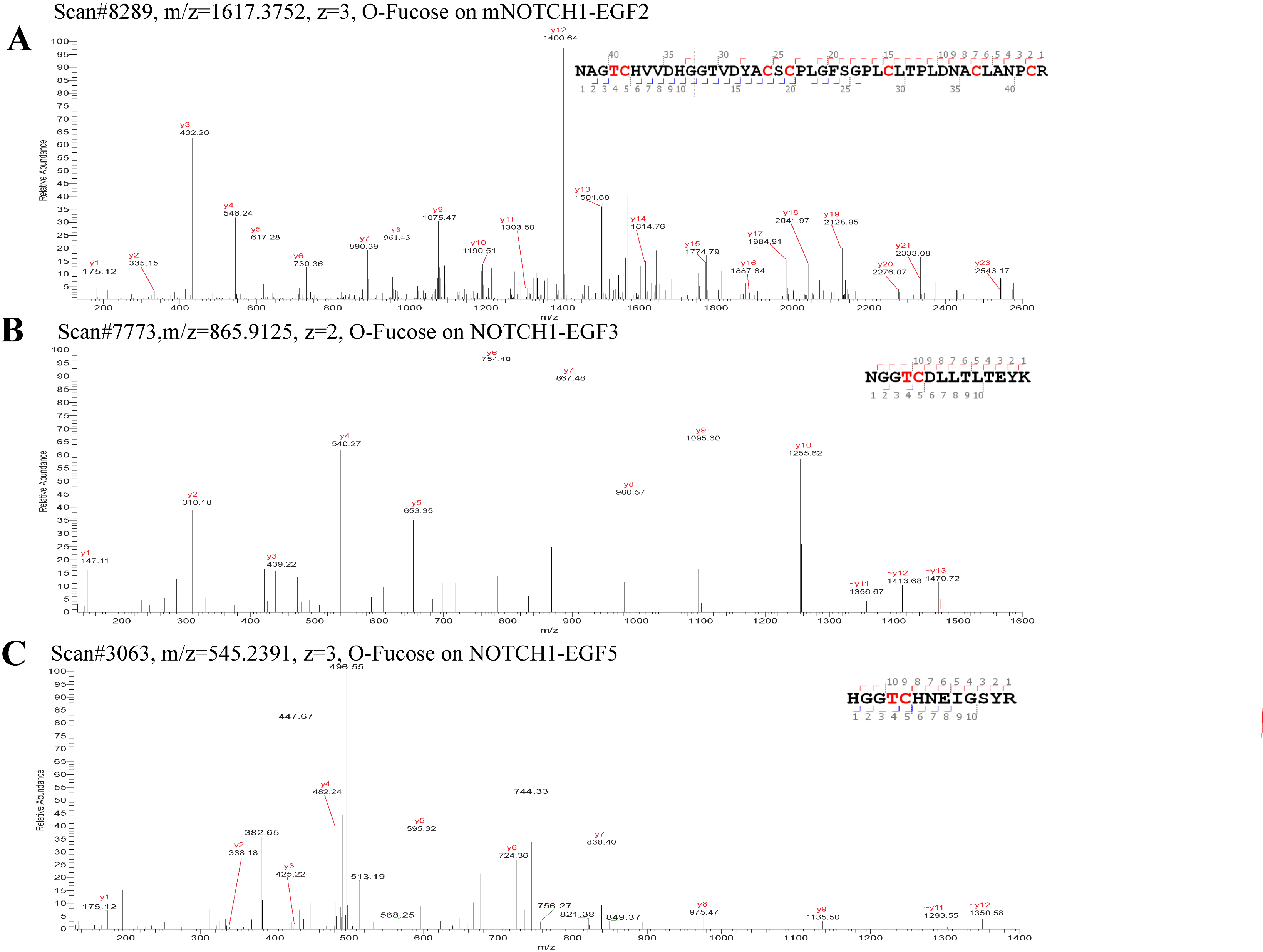


**Figure S2. Representative MS/MS spectra of O-fucosylated peptides of mouse NOTCH1 EGF repeats 1-5 expressed in wild type HEK293T cells. A.** MS/MS spectrum of the peptide with O-fucose monosaccharide on EGF2. **B.** MS/MS spectrum of the peptide with O-fucose monosaccharide on EGF3. **C.** MS/MS spectrum of the peptide with O-fucose monosaccharide on EGF5. The y Ions are indicated in red font. ~ indicates ions that lost fucose in the gas phase during fragmentation. This figure is related to Figure 2C.


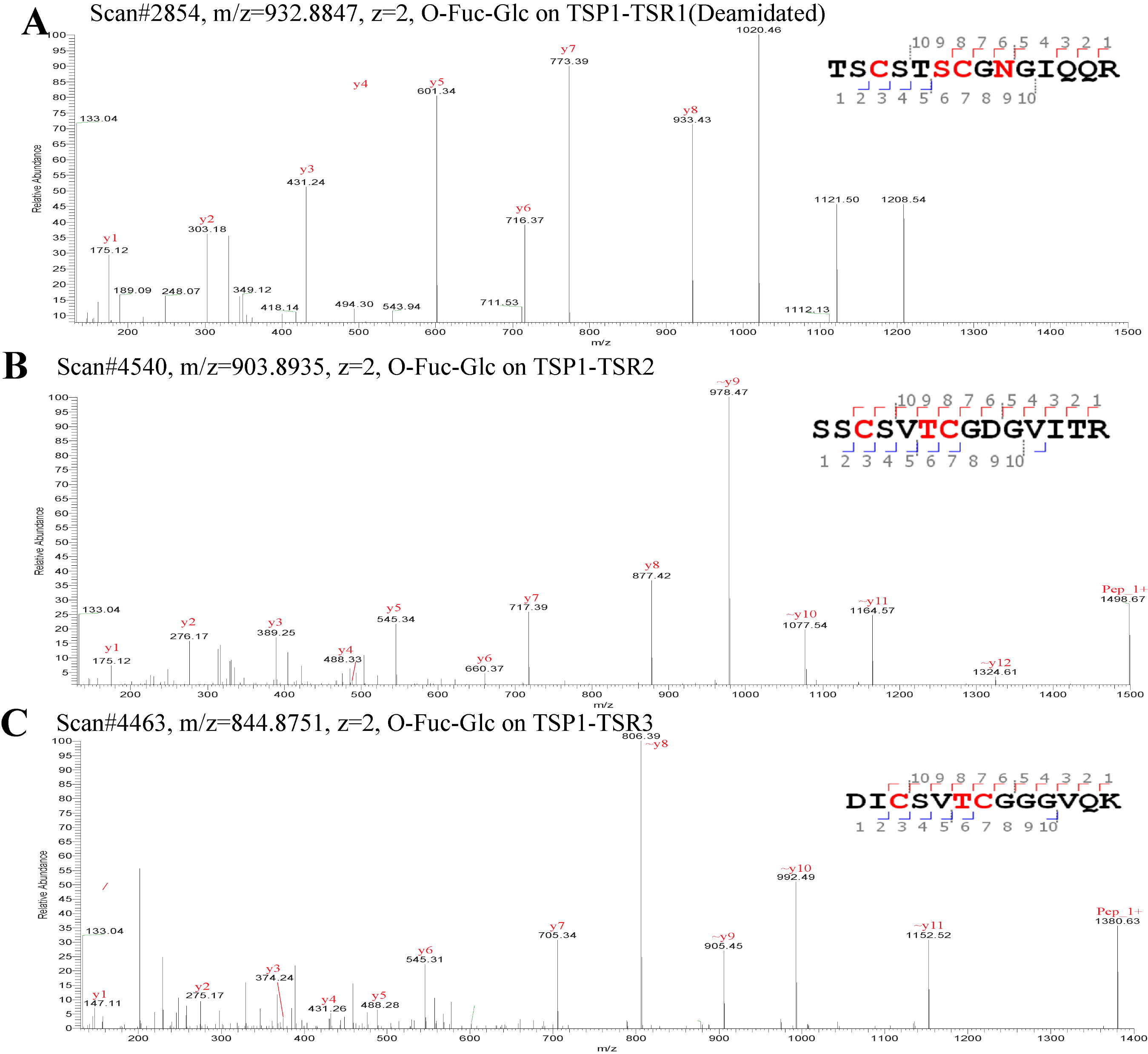


**Figure S3. Representative MS/MS spectra of O-fucosylated peptides of human TSP1 TSR repeats 1-3 expressed in wild type HEK293T cells. A.** MS/MS spectrum of the peptide with O-fucose monosaccharide on TSR1. **B.** MS/MS spectrum of the peptide with O-fucose monosaccharide on TSR2. **C.** MS/MS spectra of the peptide with O-fucose monosaccharide on TSR3. The y Ions are indicated in red font. ~ indicates ions that lost fucose-glucose in the gas phase during fragmentation. This figure is related to Figure 2C.


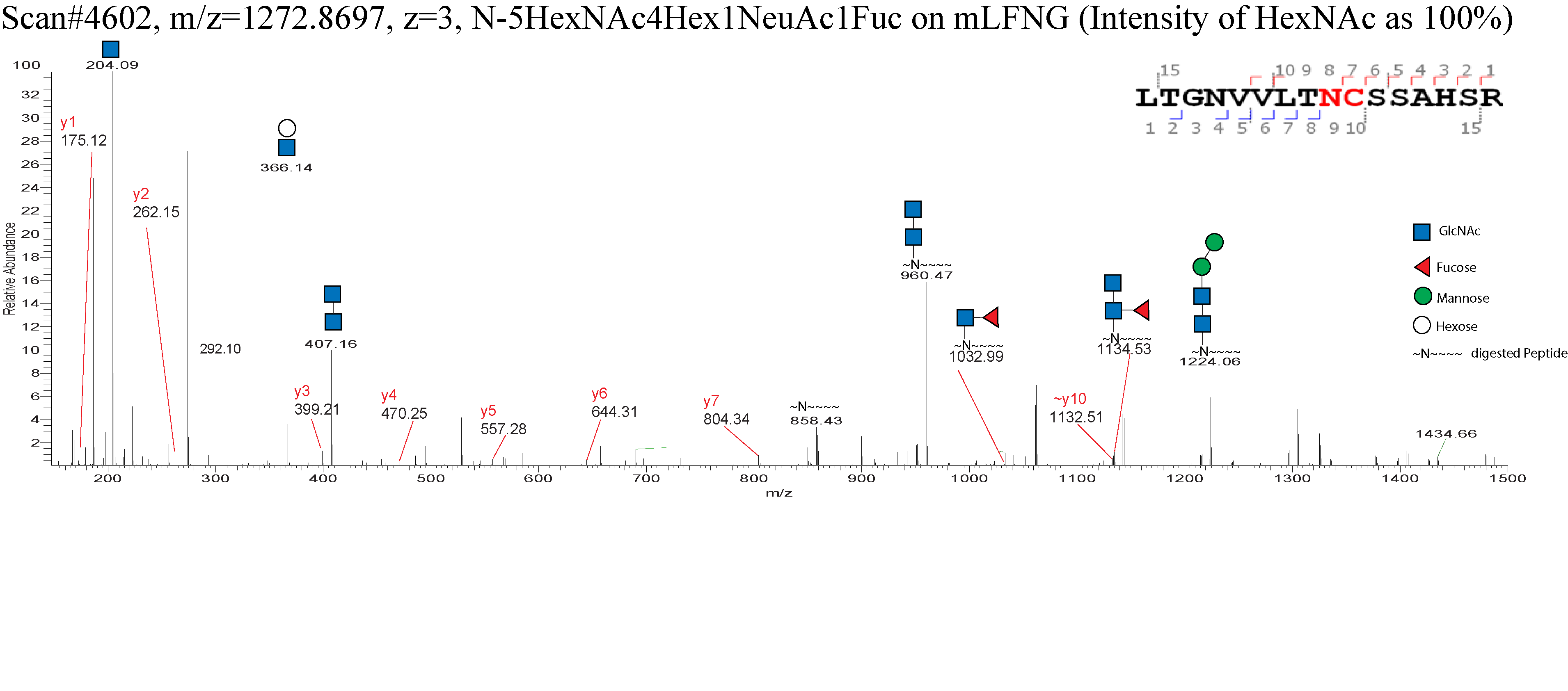


**Figure S4. Representative MS/MS spectra of a fucosylated N-glycan peptide of mouse LFNG expressed in wild type HEK293T cells.** MS/MS spectrum of the peptide modified with a 5HexNAc4Hex1NeuAc1Fuc N-glycan in mouse LFNG. The y Ions are indicated in red font, ~ indicates ions that lost an N-Glycan in the gas phase during fragmentation. Peptide ions modified with sugars or sugar oxonium ions are shown with symbols in the key. This figure is related to Figure 2D.

**Figure S5**


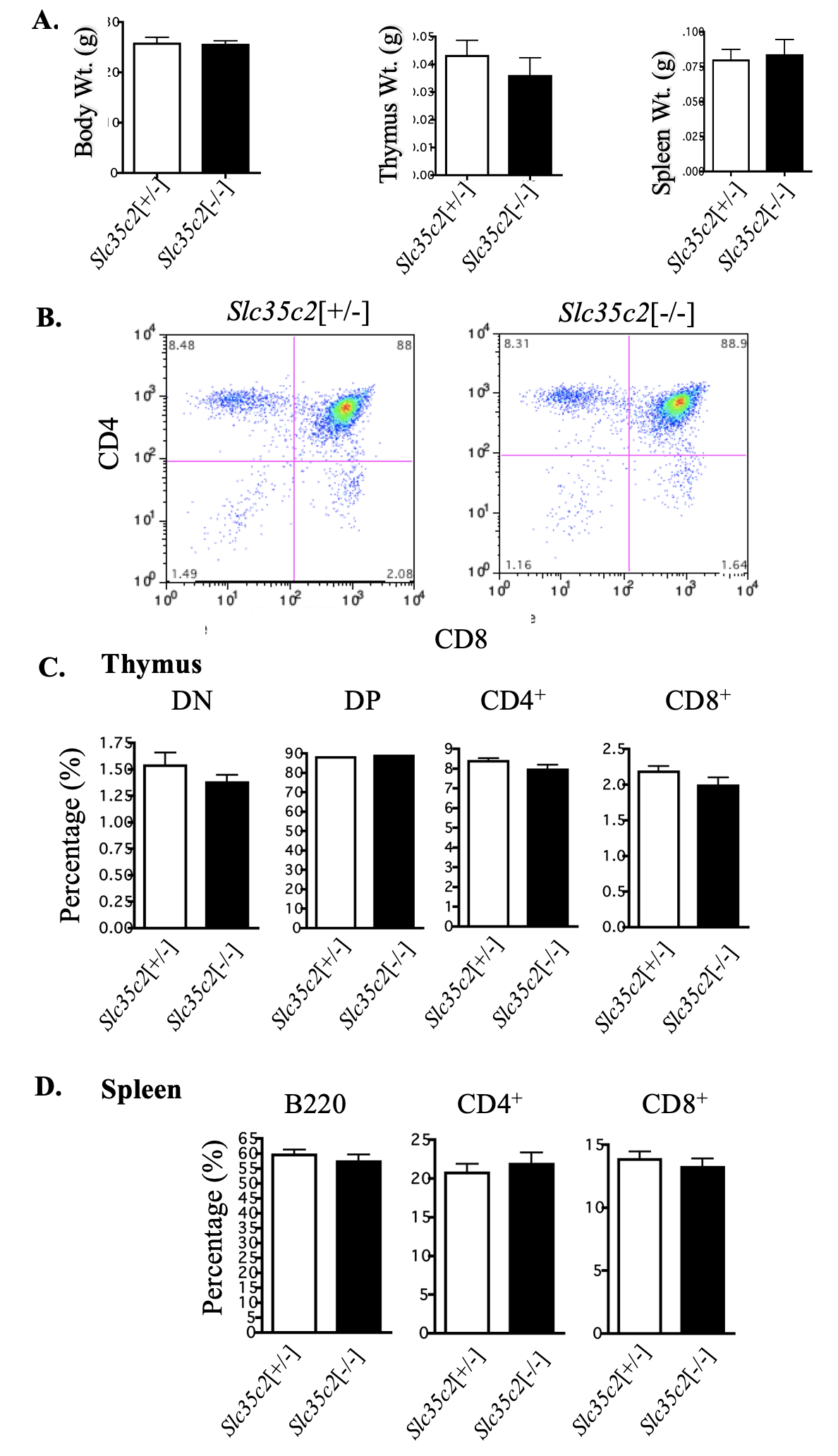


**Figure S5. T and B cell development in *Slc35c2* null mice*.* A.** Body weight, thymus weight,

and spleen weight in 7-week *Slc35c2*[+/-] (n=5) and *Slc35c2*[-/-] (n=5) mice. **B.** Flow cytometry

of thymocytes from the same mice using anti-CD4 and anti-CD8 antibodies. **C.** Frequency percent of CD4/CD8 double negative (DN), CD4/CD8 double positive (DP), CD4 and CD8 single positive (SP) cells in thymocytes. **D.** Frequency percent of B220+ B cells, CD4^+^ and CD8^+^ T cells in spleen from the same mice. Error bars represent SEM. Comparisons by unpaired, two-tailed Student’s t-test did not reveal differences of *p*<0.05.

**Figure S6**


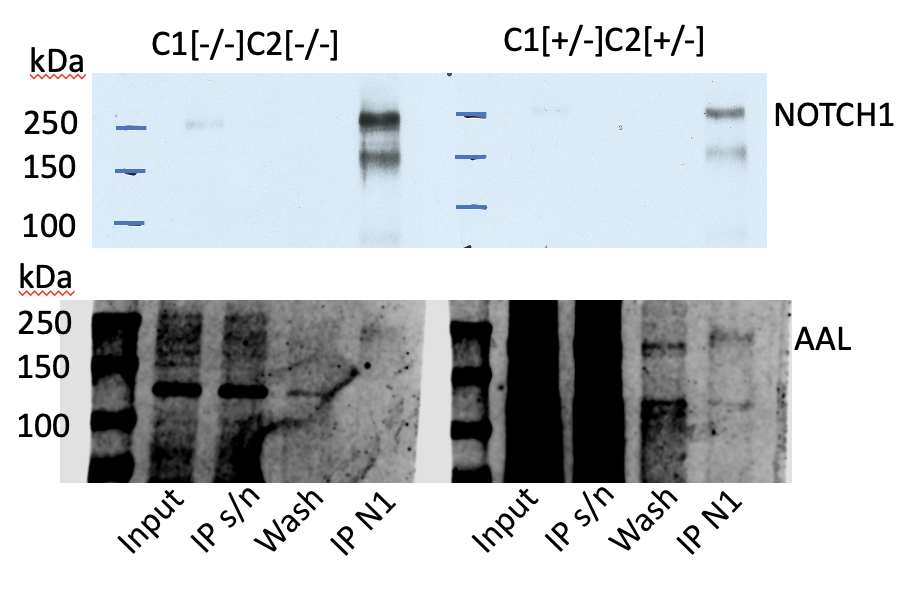


**Figure S6**. Fucosylation of NOTCH1 from lung of P28 pups. Lungs from one *Slc35c1*[-/-]*Slc35c2*[-/-] mutant and one *Slc35c1*[+/-]*Slc35c2*[+/-] control pup sacrificed at day 28 were lysed and NOTCH1 was immunoprecipitated from 0.5 mg lysate by incubation with anti-NOTCH1 ECD Ab and capture on Protein G+ agarose beads as described in Methods. Fractions analyzed by western blot were original lysate (Input), supernatant after bead incubation (IP s/n), supernatant after bead wash (Wash) and immunoprecipitate (IP N1). Molecular weight marker proteins were run in the first lane of each gel. The top gels were incubated with anti-NOTCH1 ECD Ab and the lower gels were incubated with AAL-biotin to detect fucose. This figure is related to Figure 5.

| Gene | Name | Use | 5”modification | Sequence | Reference |
| --- | --- | --- | --- | --- | --- |
| SLC35C1 | SLC35C1_EcoRI_F | Cloning | KOZAK seq | CCGCCGGAATTCGCCACCATGAATAGGGCCCCTCTGAAGCG | NM_018389.5 |
|  | SLC35C1_EcoRV_R | Cloning |  | CCGCCGGATATCTCACACCCCCATGGCGCTCTTCTC | NM_018389.5 |
| SLC35C2 | SLC35C2_EcoRI_F | Cloning | KOZAK seq | CCGCCGGAATTCGCCACCATGGGGAGGTGGG CCCTCGAT | NM_173179.4 |
|  | SLC35C2_EcoRV_R | Cloning |  | CCGCCGGATATCTCACTGCTGCCCCTGGGCCACAAAG | NM_173179.4 |
| SLC35C1 | SLC35C1_gRNA1_F | gRNA |  | GATCGCTGCCTCAAGTACGTCGGTG |  |
|  | SLC35C1_gRNA1_R | gRNA |  | AAACCACCGACGTACTTGAGGCAGC |  |
| SLC35C1 | SLC35C1_gRNA3_F | gRNA |  | GATCGGGCATTGCAGATCGCGCTGG |  |
|  | SLC35C1_gRNA3_R | gRNA |  | AAACCCAGCGCGATCTGCAATGCCC |  |
| SLC35C2 | SLC35C2_gRNA2_F | gRNA |  | GATCGCTGGGGGCCTCGTTCATCGG |  |
|  | SLC35C2_gRNA2_R | g RNA |  | AAACCCGATGAACGAGGCCCCCAGC |  |
| SLC35C2 | SLC35C2_gRNA7_F | gRNA |  | GATCGGGTGCAGCATCGTCATGAAG |  |
|  | SLC35C2_gRNA7_R | gRNA |  | AAACCTTCATGACGATGCTGCACCC |  |
| SLC35C1 | SLC35C1_gRNA1-3_F | Genotype |  | GCCCTGGACTCCAGGGAATCA | NG_009875.1 |
|  | SLC35C1_gRNA1-3_R | Genotype |  | GGTCTGCTTGAGCAGCAGGTA | NG_009875.1 |
| SLC35C2 | SLC35C2_gRNA2_F | Genotype |  | ACCCCAGGCTCCAAATGTGAT | NC_000020.11 |
|  | SLC35C2_gRNA2_R | Genotype |  | CCAGGAACATGAGTGGCTGCA | NC_000020.11 |
| SLC35C2 | SLC35C2_gRNA7_F | Genotype |  | TGGCTGCCACCTGTCTTCGTGA | NC_000020.11 |
|  | SLC35C2_gRNA7_R | Genotype |  | GGCACGCAGTGCACAAGAGGCA | NC_000020.11 |

**Table S1. Primers and guide RNAs for HEK293T experiments**

Oligonucleotides used to generate cDNA of *SLC35C1* or *SLC35C2* from wild type HEK293T cDNA, to make guide RNAs (gRNA) for CRISPR-CAS9 KO plasmids, or to amplify genomic DNA (Genotype).

**Table S2. Defective formation of thoracic vertebrae in ~E18 embryos**

| **Genotype**  **(n)** | **Mouse** | **Thoracic Vertebrae** | |  |  |  |  |
| --- | --- | --- | --- | --- | --- | --- | --- |
|  |  | T9 | T10 | | T11 | T12 | T13 |
| *C1*[-/-]*C2*[+/+] (9) | 601-1 | split | misshaped | | misshaped | split | misshaped |
|  | 601-4 |  |  | |  | hole left | misshaped |
|  | 601-6 | misshaped | misshaped | | hole right |  |  |
|  | 603-2 | misshaped | split | | 11 - 12 fused left | split | split |
|  | 603-3 |  | misshaped | | hole right | split | misshaped |
|  | 603-6 |  | misshaped | | hole left | misshaped | misshaped |
|  | 607-1 |  | misshaped | | split | misshaped |  |
|  | 607-5 |  | misshaped | | misshaped | misshaped |  |
|  | 614-8 | split | misshaped | | misshaped | split | misshaped |
| *C1*[-/-]*C2*[+/-] (3) | 818-1 | unformed | misshaped | | misshaped | misshaped | misshaped |
|  | 865-1 |  | misshaped | | misshaped | misshaped |  |
|  | 866-3 | misshaped | split | | split | split | split |
| *C1*[-/-]*C2*[-/-] (5) | 790-1 |  |  | | misshaped | split | hole right |
|  | 790-2 |  | misshaped | | split | misshaped | misshaped |
|  | 790-3 |  | misshaped | | misshaped | split | misshaped |
|  | 865-3 |  | misshaped | | split | split | misshaped |
|  | 866-4 |  | misshaped | | split |  |  |

There were no defects in T1-T8, designated by a blank cell. Genotypes with no defect in any of the 13 thoracic vertebrae were C1+/+C2+/- (n=7), C1+/+C2-/- (n=8), C1+/-C2+/- (n=7), C1+/-C2-/- (n=10).

**Table S3. Defective formation of lumbar vertebrae in ~E18 embryos**

| **Genotype**  **(n)** | **Mouse** | **Lumbar** | **Vertebrae** |  |  |  |
| --- | --- | --- | --- | --- | --- | --- |
|  |  | L1 | L2 | L3 | L4 | L5 |
| *C1*[-/-]*C2*[+/+] (9) | 601-1 | misshaped | split | split | unshaped | misshaped |
|  | 601-4 | misshaped | misshaped | misshaped | split | misshaped |
|  | 601-6 | misshaped | misshaped | misshaped | misshaped | misshaped |
|  | 603-2 | hole left | hole right | split | split | split |
|  | 603-3 | misshaped | split | misshaped | misshaped | misshaped |
|  | 603-6 | misshaped | misshaped | misshaped | split | misshaped |
|  | 607-1 | misshaped | misshaped | misshaped | misshaped | split |
|  | 607-5 | misshaped | misshaped | misshaped | split | misshaped |
|  | 614-8 | misshaped | split | split | misshaped | misshaped |
|  |  |  |  |  |  |  |
| *C1*[-/-]*C2*[+/-] (3) | 818-1 | hole right | unformed, split | misshaped | misshaped |  |
|  | 865-1 | hole right | unformed, split | misshaped | misshaped |  |
|  | 866-3 | misshaped | misshaped | misshaped | misshaped | split |
|  |  |  |  |  |  |  |
| *C1*[-/-]*C2*[-/-] (5) | 790-1 | misshaped | misshaped | misshaped | misshaped | split |
|  | 790-2 | split, hole right | misshaped | split,hole right | split, hole left | misshaped |
|  | 790-3 | misshaped | misshaped | split | misshaped | misshaped |
|  | 865-3 | misshaped | misshaped | split | misshaped | split |
|  | 866-4 | misshaped | misshaped | split | unshaped | misshaped |

There were no defects in the L6 vertebra. Other lumbar vertebrae with no defects designated by a blank cell. Genotypes with no defects in any lumbar vertebrae were C1+/+C2-/- (n=8), C1+/-C2-/- (n=9), C1+/-C2+/- (n=7), and C1+/+C2+/- (n=7). Mouse 684-5 (C1+/-C2-/-) had a misshaped 4^th^ lumbar vertebra.

**Table S4. Defective formation of sternum, ribs and tail in ~E18 embryos**

| **Genotype** | **Mouse** | |  |  | **Ribs** |  |  |  | **Sternum** | **Tail** |
| --- | --- | --- | --- | --- | --- | --- | --- | --- | --- | --- |
| (n) |  | **R1 - R8** | | **R9** | **R10** | **R11** | **R12** | **R13** | **S1-S4** | **C1-C17** |
| *C1*[-/-]*C2*[+/+] (9) | 601-1 | R4, R5  assym | | R9  curved | L10  curved |  |  | L13, R13 broken | S3-S4  S4-C1  assym |  |
|  | 601-4 | R4, R5  assym | |  | R10 curved | R11  sharp curved | L12 curved | R13 sharp  curved ,broken  L13 missing | S4-C1  assym |  |
|  | 601-6 |  | |  |  |  |  | L13 broken |  |  |
|  | 603-2 | R4, R5  assym | |  | L10, L11 fused at root |  | L12 curved, R12 missing | L13, R13 missing | S3-S4  S4-C1  assym |  |
|  | 603-3 |  | |  | R10 curved | R11 curved |  | L13 curved, R13 broken |  | C8-C9 fused |
|  | 603-6 | R4, R5  assym | | R9  curved | L10,11 fused at root, L10 curved | normal | L12 curved | L13 missing | S3-S4  S4-C1 |  |
|  | 607-1 |  | |  |  | R11 curved |  | R13 curved |  | C8-C9 fused |
|  | 607-5 |  | |  |  | L11 curved |  | R13 broken |  |  |
|  | 614-8 | R4, R5  assym | | L9  curved | L10, R10 curved |  |  | L13, R13 broken | S3-S4  S4-C1  assym | C1-C2  broken |
| *C1*[-/-]*C2*[+/-] (3) | 818-1 | R1 - R2  fused | | R9 - R10 fused at root | R10 sharp curved, L10 bent | R11 broken, L11 bent | L12, R12  bent | L13 curved, R13 missing cartilage | mess |  |
|  | 865-1 | R4, R5  assym | |  |  | R11 curved |  |  | S3-S4,  S4-C1  assym |  |
|  | 866-3 |  | |  | R10 curved |  |  | R13 curved |  |  |
| *C1*[-/-]*C2*[-/-]  (5) | 790-1 |  | |  |  |  | R12-R13 fused | L13 missing |  | C5, C6 fused |
|  | 790-2 |  | |  | R10 curved | R11 curved |  |  |  | C5, C6 fused |
|  | 790-3 | R1  bifurc | |  |  | R11 curved | R13 curved | L13 missing |  | C5, C6 fused |
|  | 865-3 |  | |  | R10 curved | R11 curved |  |  |  | C5, C6 fused |
|  | 866-4 |  | |  | R10 curved |  | R12&13 fused | L13 missing |  | C5, C6 fused |

A blank cell signifies no defect. Within the table, R signifies right, L signifies left. Genotypes with no defect in sternum, ribs or tail were C1+/+C2+/- (n=7), C1+/-C2+/- (n=7), C1+/-C2-/- (n=10), C1+/+C2-/- (n=8). assym, assymetrical; bifurc, bifurcated.

**Table S5.** **Progeny of backcrossed *Slc35c1* and *Slc35c2* mutant mice**

| **Cross** | **Litters** | **No.**  **Progeny (Age)** | **Observed**  **(Frequency %)** | **Expected**  **(Frequency %)** | ***Chi***  **Squared**  **(*p*)** |
| --- | --- | --- | --- | --- | --- |
|  |  |  | **+/+ +/- -/-** | **+/+ +/- -/-** |  |
| **C57BL/6J** |  |  |  |  |  |
| *C2*[+/-] X *C2*[+/-] | 12 | 93 (P8) | 28 55 17 | 25 50 25 | 0.18 |
|  |  |  |  |  |  |
| *C2*[+/-] X *C2*[-/-] | 6 | 47 (P8) | 50 50 | 50 50 | 1.0 |
|  |  |  |  |  |  |
| *C1*[+/-]*C2*[-/-] X | 12 | 76 (P8) | *C1*  28 67 5 | 25 50 25 | **<0.0001** |
| *C1*[+/-]*C2*[-/-] |  |  | *C2* 100 |  | N/A |
|  |  |  |  |  |  |
| *C1*[+/+]*C2*[+/-] X | 3 | 18 (E18) | *C1* 61 39 | 50 50 | 0.35 |
| *C1*[+/-]*C2*[-/-] |  |  | *C2* 50 50 | 50 50 | 1.0 |
|  |  |  |  |  |  |
| *C1*[+/-]*C2*[+/-] X | 3 | 14 (E18) | *C1*  21 50 29 | 25 50 25 | 0.80 |
| *C1*[+/-]*C2*[-/-] |  |  | *C2* 14 86 | 50 50 | **0.008** |
|  |  |  |  |  |  |
| **129SvJ** |  |  |  |  |  |
| *C1*[+/-]C2[+/-] X | 10 | 58 (P8) | *C1* 26 59 15 | 25 50 50 | 0.30 |
| *C1*[+/-]*C2*[+/-] |  |  | *C2* 9 57 34 | 25 50 50 | **0.013** |
|  |  |  |  |  |  |
| *C1*[+/-]*C2*[+/-] X | 4 | 18 (P8) | *C1* 28 55 17 | 25 50 50 | 0.78 |
| *C1*[+/-]*C2*[-/-] |  |  | *C2* 44 56 | 50 50 | 0.64 |

N/A, not applicable

**Table S6. Skeletal defects in *Slc35c1:Slc35c2* null neonates after backcrossing to 129X1/SvJ**

| **Mouse** | **Lumbar** | **Ribs** |
| --- | --- | --- |
| *C1*[-/-]*C2*[-/-] |  |  |
| 19-3 |  | R11-R13 bent |
| 19-4 | L3 split, L5, L6 misshaped |  |
| 19-5 |  | R13 fused |
| 21-8 | L5 split, L6 misshaped | R13 fused |
| 21-9 | L4 split | R11-R13 bent |

Skeletons of P0 pups from crosses between *Slc35c1*[+/-] and *Slc35c2*[+/-] mice that had been backcrossed 8 times to 129X1/SvJ mice were characterized for the following genotypes: C1+/-C2+/- (n=9), C1+/+C2-/- (n=4), C1+/-C2-/- (n=5) and C1-/-C2-/- (n=5) where C1 is *Slc35c1* and C2 is *Slc35c2.* The only abnormalities observed were in C1-/-C2-/- skeletons lumbar region and ribs as tabulated above, except for 1 C1+/+C2-/- mouse that had a misshaped L4.

| Genotyping Primers | | | |
| --- | --- | --- | --- |
| Gene | Name | Sequence | |
| *Slc35c1* | PS1250 | *ACTTAAGCTTATGAACAGGGCGCCTCTGAA* | |
|  | PS1354 | *GTGAGCAGGGCATAGAAGGAAGTGGTC* | |
|  | PS1355 | *GGATCCCGTCGACGGTATCGATAAGC* | |
|  |  |  | |
| *Slc35c2* | PS1115 | *GCCTGGTCCTTCTATACTACTGCTTCTCCATAG* | |
|  | PS1324 | *GGAAGCTCTGTGAAGCCCAAAGACG* | |
|  | PS1325 | *GGACGGCGAGCTCGAATTGATCC* | |
|  |  |  | |
| qRT-PCR Primers | | | |
| Gene | **Name** | **Sequence** | |
| *Slc35d1* | Forward | *CTAAATATCAGTATTGCTC* | |
|  | Reverse | *AGCTTGTTACTGGCCT* | |
| *Slc35d2* | Forward | *CAGAGATGGAGGAGCCCAAC* | |
|  | Reverse | *TGGTGAGGGAAAACCGTATGT* | |
| *Slc35e1* | Forward | *AGTCCTGCTAGCCACTGTCA* | |
|  | Reverse | *GAGTCCCTCAACACCTTTTTGG* | |
| *Slc35e2* | Forward | *GACGTTCAGTGTCGCCAGTA* | |
|  | Reverse | *GCTGTGACCAGACTCTGCAT* | |
| *Gapdh* | Forward | *AAGGTCATCCCAGAGCTGAA* |  |
|  | Reverse | *CTGCTTCACCACCTTCTTGA* |  |
| *Actb* | Forward | *TTCTACAATGAGCTGCGTGTG* |  |
|  | Reverse | *GGGGTGTTGAAGGTCTCAAA* | |

**Table S7. Primers for mouse genotyping and qRT-PCR of lung and liver transcripts**

Genotyping by PCR of genomic DNA used the following primer pairs to obtain the designated products: *Slc35c1* WT: PS1250 + PS1354 = 530 bp; *Slc35c1* Del: PS1250 + PS1355 = 460 bp;

*Slc35c2* WT: PS1115 + PS 1324 = 390 bp; *Slc35c2* Del: PS1115 + PS1325 = 230 bp.

WT, wild type allele; Del, deleted allele.
